# Advances in anion binding and sensing using luminescent lanthanide complexes†

**DOI:** 10.1039/d0sc05419d

**Published:** 2021-01-26

**Authors:** Samantha E. Bodman, Stephen J. Butler

**Affiliations:** Department of Chemistry, Loughborough University Epinal Way, Loughborough LE11 3TU UK S.J.Butler@lboro.ac.uk

## Abstract

Luminescent lanthanide complexes have been actively studied as selective anion receptors for the past two decades. Ln(iii) complexes, particularly of europium(iii) and terbium(iii), offer unique photophysical properties that are very valuable for anion sensing in biological media, including long luminescence lifetimes (milliseconds) that enable time-gating methods to eliminate background autofluorescence from biomolecules, and line-like emission spectra that allow ratiometric measurements. By careful design of the organic ligand, stable Ln(iii) complexes can be devised for rapid and reversible anion binding, providing a luminescence response that is fast and sensitive, offering the high spatial resolution required for biological imaging applications. This review focuses on recent progress in the development of Ln(iii) receptors that exhibit sufficiently high anion selectivity to be utilised in biological or environmental sensing applications. We evaluate the mechanisms of anion binding and sensing, and the strategies employed to tune anion affinity and selectivity, through variations in the structure and geometry of the ligand. We highlight examples of luminescent Ln(iii) receptors that have been utilised to detect and quantify specific anions in biological media (*e.g.* human serum), monitor enzyme reactions in real-time, and visualise target anions with high sensitivity in living cells.

## Abstract 2

This minireview highlights advances in anion binding and sensing using luminescent lanthanide(iii) complexes.

## Graphical abstract

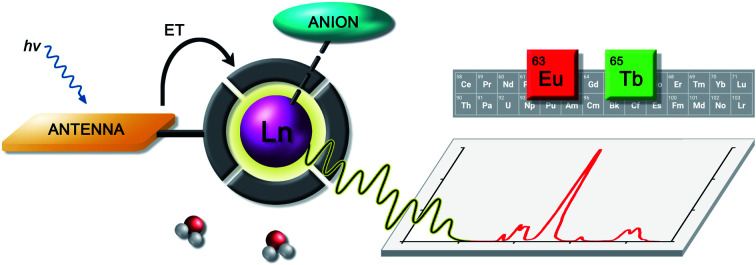

## Introduction

The development of luminescent lanthanide(iii) complexes for the purpose of binding and sensing anions in water has advanced considerably in recent years. The creation of Ln(iii)-based anion receptors is driven by the need for new sensing and imaging tools for biological, clinical and drug discovery research.^1–3^ For example, an emissive Ln(iii) receptor capable of binding bicarbonate (HCO_3_^−^) selectively could be used to image spatial-temporal HCO_3_^−^ dynamics in living cells using fluorescence microscopy,^4^ potentially aiding the diagnosis and treatment of diseases such as renal disease and glaucoma.^5^ A Ln(iii) receptor that binds adenosine diphosphate (ADP) selectively could allow real-time analysis of kinase enzyme activity by monitoring the production of ADP,^6^ thereby providing a convenient luminescence assay for high throughput screening of potential kinase inhibitors for the treatment of cancer.^7,8^ Many other sensing and bioimaging applications can be envisaged for Ln(iii) receptors,^9–11^ which serve to enhance the technologies available to biological and medical scientists.

**Fig. 1:**
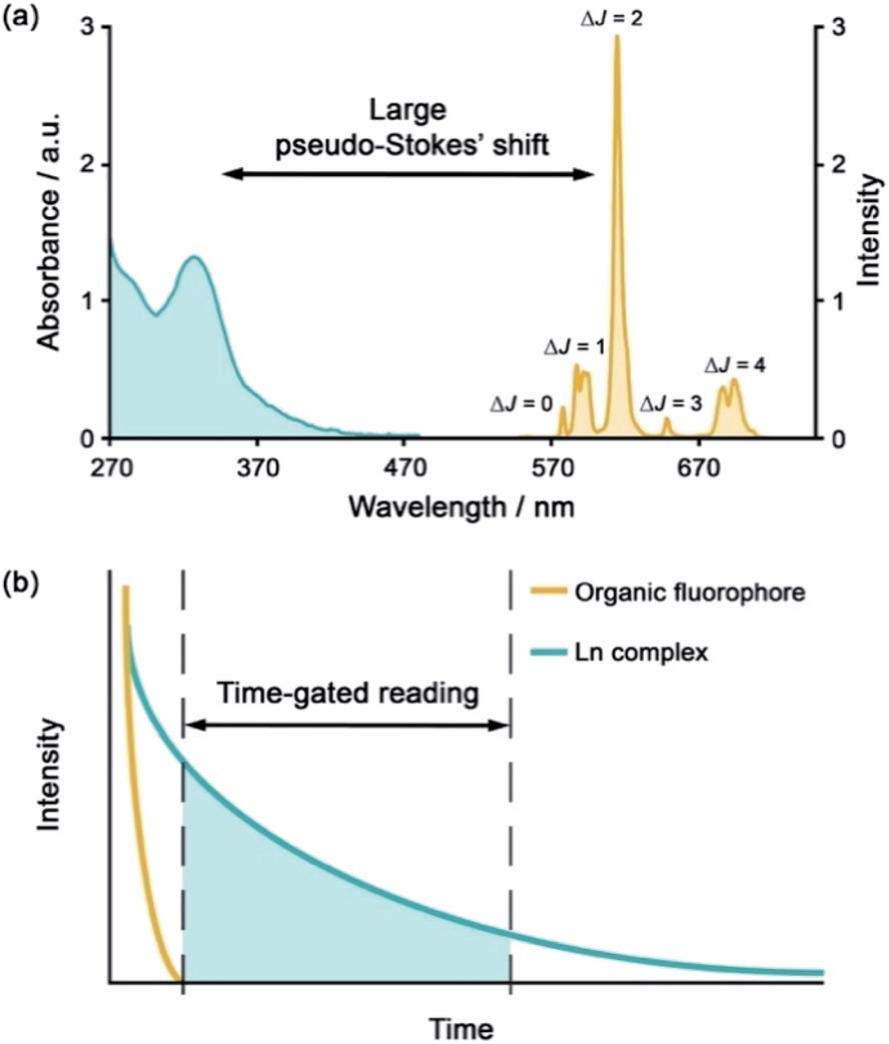
(a) Representative absorption and emission spectra of Eu(iii) complexes highlighting the large pseudo-Stokes'shift; (b) illustration of time-gated detection to eliminate short-lived autofluorescence arising from biomolecules in the sample.

Emissive Ln(iii) complexes, particularly of europium(iii) and terbium(iii) which emit in the red and green spectral region respectively, offer unique photophysical properties that are very valuable for anion sensing in biological media.^12,13^ Firstly, they possess long luminescence lifetimes (up to milliseconds) that enable time-gated or time-resolved measurements to distinguish the lanthanide-centred emission from the short-lived autofluorescence of biological samples.^1,14,15^ Time-gating methods offer enhanced signal-to-noise and very low limits of anion detection (Fig. 1). Secondly, they have sharp line-like emission spectra, which can allow ratiometric analysis by measuring the change in intensity of one emission band relative to the almost stationary intensity of a second band (*e.g.*, comparing the hypersensitive Δ*J* = 2 emission band of Eu(iii) around 615 nm with the Δ*J* = 1 emission band around 590 nm).^16^ Thirdly, by careful design of the surrounding organic ligand, Ln(iii) complexes capable of rapid and reversible anion binding can be created, providing a luminescence response that is fast and sensitive, offering the high spatial resolution required for biological imaging applications.^3,13^

Over the last two decades, a variety of Ln(iii) complexes have been developed for binding and sensing anions, cations and biomolecules in aqueous media, and these have been summarized effectively in some comprehensive reviews.^2,11,17–19^ Recently, a few examples of anion responsive Ln(iii) coordination polymers have emerged, which may be explored as potential sensing materials.^20,21^ However, examples of Ln(iii) complexes that exhibit high selectivity for a target anion are still relatively rare. This review focuses on recent examples of discrete, water-soluble Ln(iii) complexes that display sufficiently high anion selectivity to be utilised in biological or environmental sensing applications. We distinguish the mechanisms of anion binding and discuss how selectivity can be tuned through variations in ligand structure and geometry. This review is not comprehensive, and a particular focus is given to Eu(iii) and Tb(iii) receptors that can detect anions in biological media (*e.g.* human serum), monitor enzyme reactions involving anions in real-time, or visualise fluctuations of target anions in living cells using fluorescence microscopy.

### Lanthanide receptor design considerations

Anion recognition in water is challenging for several reasons; anions have a range of geometries, they may be pH sensitive (*e.g.*, H_2_PO_4_^−^/HPO_4_^2−^, HCO_3_^−^/CO_3_^2−^), and have high hydration energies.^22,23^ Molecular receptors that utilise coulombic attraction or strong metal–ligand interactions are required to overcome the strong interactions between an anion and its hydration sphere. Selectivity for a target anion may be achieved by integrating additional binding sites within the receptor, creating a structured binding pocket with high geometric complementarity.^24,25^ The nature and abundance of potentially interfering species in the aqueous media under investigation must be considered, such as competing anions and cations, or complex ionic biomolecules (*e.g.* proteins, nucleic acids), as this defines the requirements for selectivity of the receptor.^2,26^

**Fig. 2:**
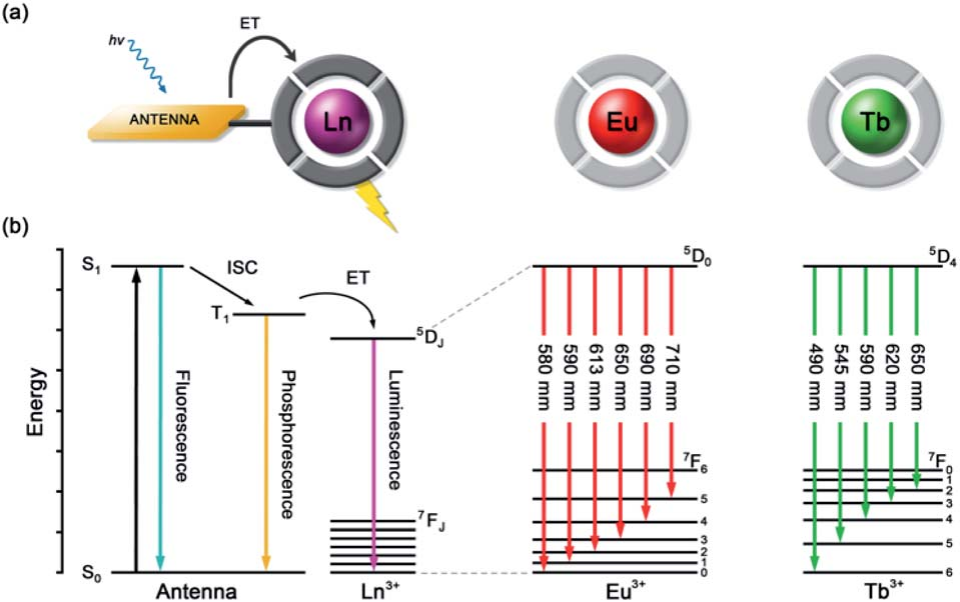
(a) Schematic depiction of the antenna effect; (b) simplified Jablonski diagram showing the common pathway leading to lanthanide sensitization, and the 4f–4f transitions of Eu(iii) and Tb(iii) complexes.

Lanthanide ions adopt coordination numbers between 8 and 10 in aqueous solution and the interaction with the surrounding ligand is predominantly electrostatic in nature.^27^ Ln(iii) complexes based on polydentate ligands (*e.g.* the azamacrocycle DO3A) offer significant scope for the design of selective anion receptors, in which the affinity and selectivity may be modulated by variations in the ligand structure, geometry and conformational flexibility, steric hindrance around the Ln(iii) ion, and the overall charge of the complex. When designing such receptors, the ligand should incorporate a sufficient number (6–8) of hard donor atoms (*e.g.* oxygen and nitrogen) to ensure high kinetic and thermodynamic stability of the Ln(iii) complex, both in the absence and presence of anions, to avoid complications arising from metal ion dissociation. In addition to the structural requirements for anion binding, to overcome the intrinsic low molar absorptivity of the lanthanides, a strongly absorbing chromophore (or antenna) should be integrated into the ligand structure, which can be excited by UV/visible light before transferring its energy to the proximal Ln(iii) ion (Fig. 2a).^28,29^ A simplified Jablonski diagram depicting the most common mechanism of sensitization of Eu(iii) or Tb(iii), *via* energy transfer from the triplet (T_1_) excited state of an absorbing antenna, is given in Fig. 2b.

### Distinct binding and signalling mechanisms

**Fig. 3:**
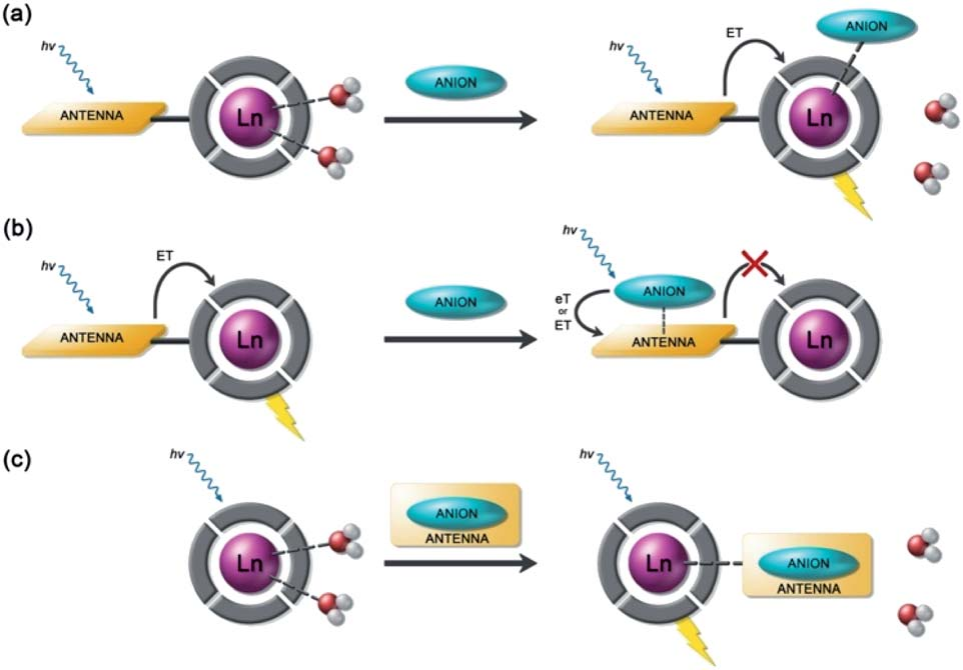
Schematic representation of different sensing mechanisms for lanthanide(iii)-based anion receptors: (a) coordination of the anion to the metal centre with displacement of inner-sphere water molecules; (b) interaction of the anion with the antenna causing electron or energy transfer, which modulates the sensitisation process, usually quenching luminescence; (c) binding of an anion that possesses an appropriate sensitiser, which ‘switches on’ luminescence.

**Fig. 4:**
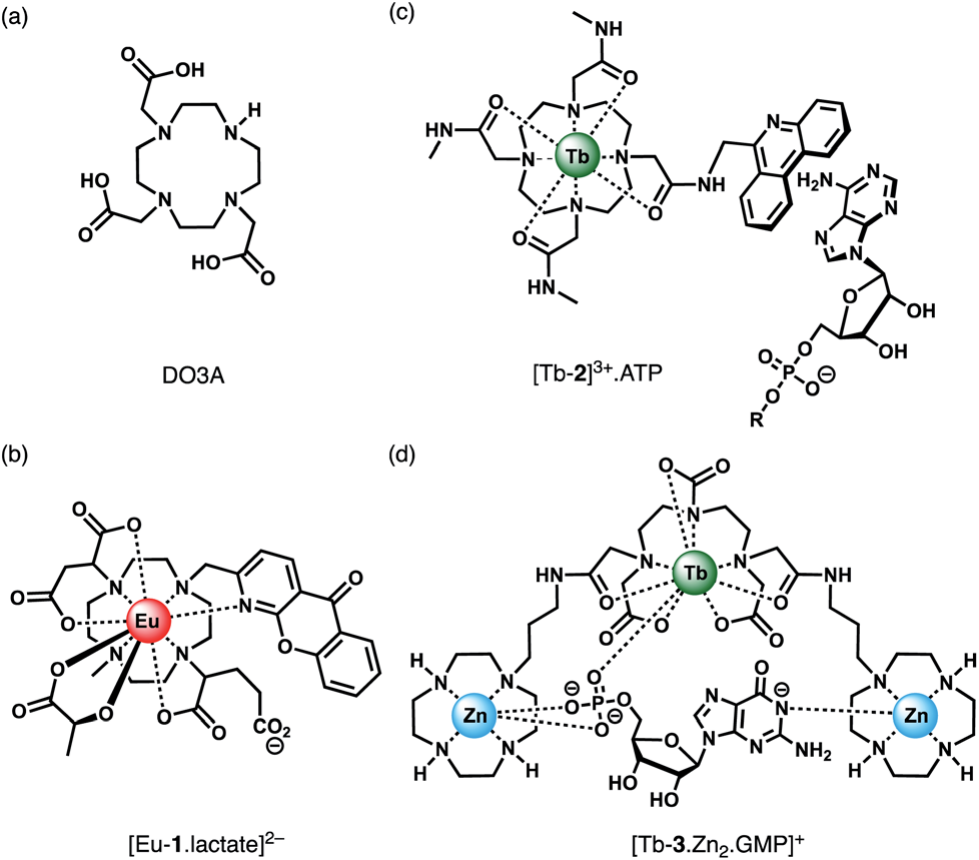
(a) Common macrocyclic ligand DO3A; (b)–(d) examples of Ln(iii) receptors that utilise different mechanisms of anion binding.

Anion binding and signalling at Ln(iii) centres may be achieved through a variety of mechanisms (Fig. 3). Anion binding may occur directly at the metal centre, involving displacement of one or more inner-sphere water molecules and variations in the Ln(iii) coordination environment, resulting in changes in the emission intensity, spectral form and lifetime of the complex (Fig. 3a). Notably, the changes in emission spectral form induced by anion binding (typically characterised by a large change in the Δ*J* = 2 emission band relative to the Δ*J* = 1 band for Eu(iii) complexes) offers the opportunity for ratiometric analysis. This attractive strategy has been utilised effectively in influential work by Parker and co-workers,^30^ wherein a range of heptadentate ligands based on DO3A (Fig. 4a) were designed to generate emissive Eu(iii) and Tb(iii) complexes with one or two available anion binding sites, occupied by water molecules in aqueous solution. The coordinated water causes efficient deactivation of the Ln(iii) excited state, *via* non-radiative energy transfer to vibrational modes of the O–H groups. Upon binding to certain anions, such as lactate, HPO_4_^2−^, HCO_3_^−^, and citrate,^30–32^ the inner sphere water is displaced, causing enhancements in emission intensity and lifetime, as well as changes in spectral shape induced by changes in the Ln(iii) coordination environment. Several of these Ln(iii) receptors have been developed into practical assays for the selective and ratiometric detection of anions in biological fluids, including lactate (Fig. 4b, [Eu-**1**]), HCO_3_^−^ and citrate.^33–35^

Alternatively, anion binding may involve a non-coordinative interaction with the antenna or other components of the ligand framework, including π–π stacking between two aromatic components, hydrogen bonding, or electrostatic interactions with an overall cationic complex (Fig. 3b). In most of these cases, anion binding causes quenching of the singlet or triplet excited state of the antenna by an energy or charge transfer mechanism (*e.g.* from an electron rich anion to an electron poor antenna), causing a decrease in Ln(iii) emission intensity. The binding interactions are typically weaker than those involving direct coordination of the anion to the Ln(iii) ion; consequently, this approach may be better suited for sensing anions present at relatively high (millimolar) concentrations. This strategy is nicely exemplified by a Tb(iii) complex developed by Pierre and co-workers, which is capable of binding ATP through a combination of electrostatic and π–π stacking interactions between the adenine group and phenanthridine antenna, resulting in quenching of Tb(iii) luminescence (Fig. 4c, [Tb-**2**]^3+^·ATP).^36^

Finally, a less common signalling mechanism involves the ‘switching on’ of Ln(iii) emission upon binding an anion that possesses an appropriate sensitising chromophore (Fig. 3c). This requires matching of the anion's triplet excited state energy with the Ln(iii) excited state, such that back energy transfer is minimal. Whilst this approach can lead to high levels of selectivity for a target anion, it is also limited in scope due to the requirement of the anion to possess a suitable chromophore. This approach was employed recently for the selective recognition of guanosine monophosphate (GMP) using a Tb(iii)–bisZn(ii) complex, which becomes emissive upon sensitisation of the Tb(iii) centre by the proximal guanine unit (Fig. 4d, [Tb-**3**·Zn_2_·GMP]^4+^).^37^

## Receptors for inorganic phosphate

Inorganic phosphate has critical roles in skeletal mineralisation, energy production/transfer and cellular signalling. Human blood levels of phosphate are maintained within a relatively narrow range, between 0.8–1.45 mM,^38^ comparable with lactate (0.5–1.0 mM) and significantly lower than HCO_3_^−^ (23–29 mM).^39^ The intracellular concentration of inorganic phosphate is higher than in serum, ranging between 1–5 mM depending on the tissue, pH and hormone levels. Elevated phosphate levels are associated with diabetes and renal disease and can cause vascular calcification, increasing the risk of stroke. In the environment, inorganic phosphate is essential in phosphorus fertilizers to support food production. However, over-use of such fertilizers has led to an increase in phosphate in surface waters, from its typical range 0.01–0.1 mM,^40^ leading to eutrophication, algal blooms and formation of aquatic dead-zones.

A receptor for the detection of phosphate in blood serum should be tuned to the millimolar range and exhibit high selectivity over HCO_3_^−^ and lactate. This is very challenging because these oxyanions intrinsically compete with phosphate for coordination at the Ln(iii) centre. Other anions present in serum, such as chloride, nitrate and sulfate, are poorly coordinating anions to Ln(iii) ions, allowing high selectivity for phosphate. The recognition of phosphate will also depend on the pH. At physiological pH, phosphate is present predominantly in the forms H_2_PO_4_^−^ and HPO_4_^2−^, in approximately a 1 : 4 ratio. A Ln(iii) receptor will normally exhibit higher affinity for phosphate when it is deprotonated at higher pH; however, the competitive binding of HCO_3_^−^/CO_3_^2−^ is also likely to occur.

**Fig. 5:**
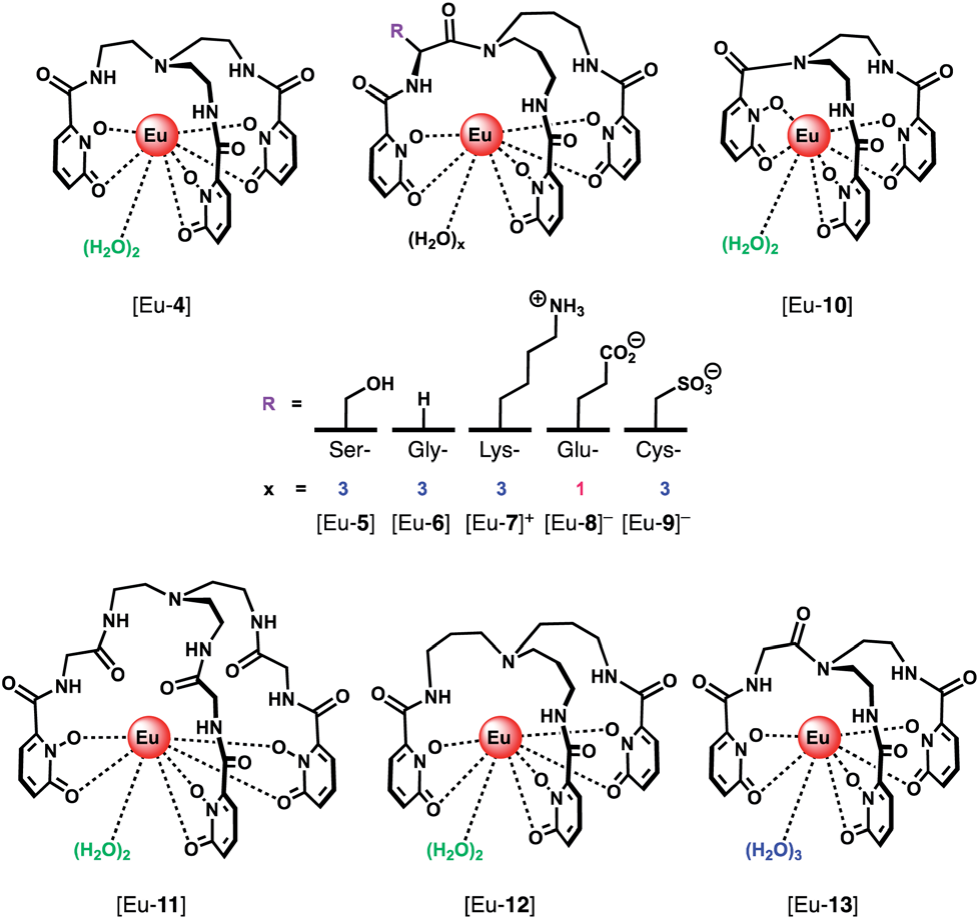
Series of tripodal Eu(iii) complexes evaluated for the selective detection of HPO_4_^2−^.

**Fig. 6:**
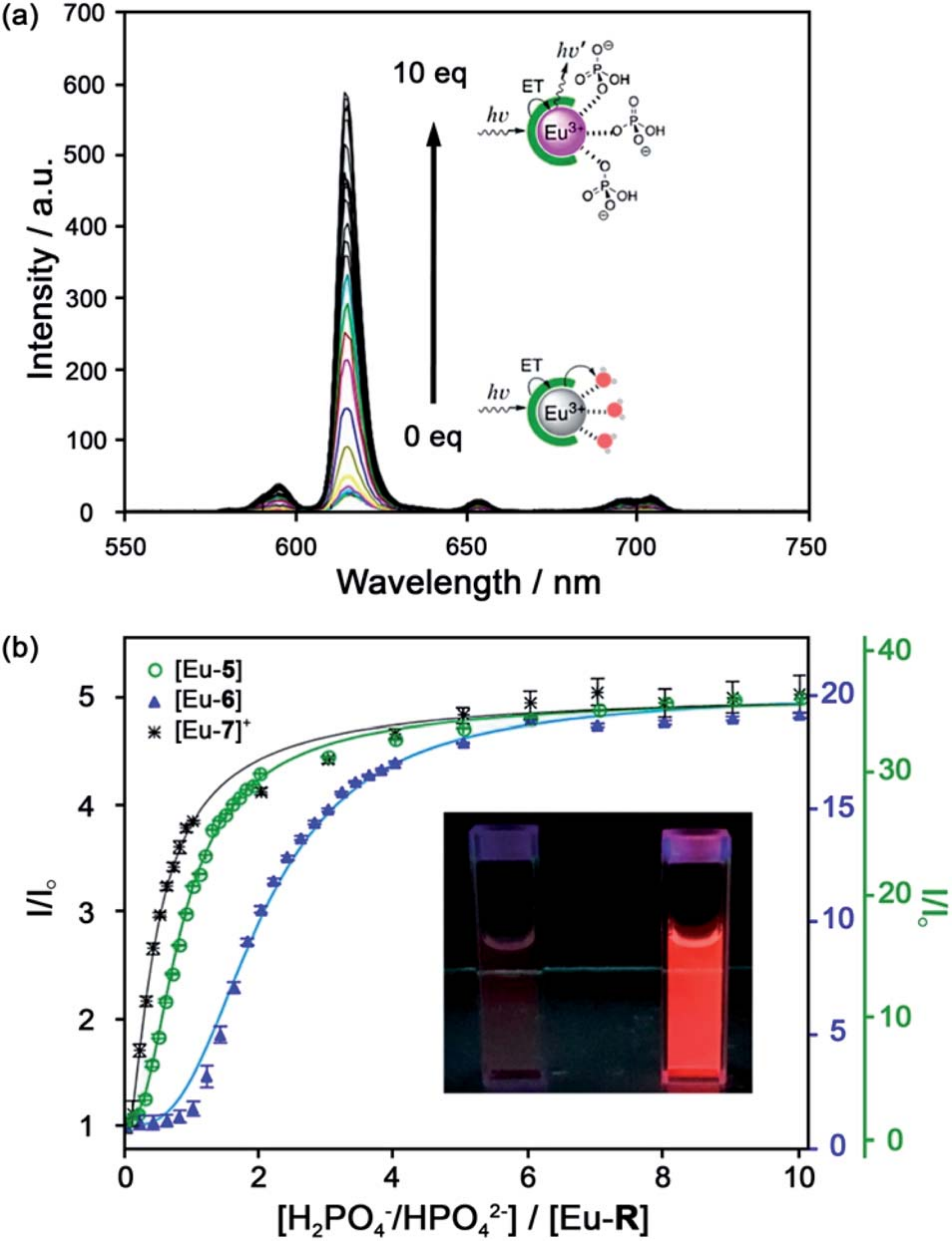
(a) Increase in time-delayed (0.1 ms) luminescence of [Eu-**6**] upon titration with HPO_4_^2−^ (H_2_O, pH 7.4); (b) luminescence titration data of [Eu-**5**], [Eu-**6**], and [Eu-**7**]^+^. Inset: Photo of [Eu-**6**]^+^ solution in the absence and presence of 10 equiv. of HPO_4_^2−^. Reproduced from ref. ^41^ with permission from the American Chemical Society, copyright 2019.

A series of Eu(iii) complexes that show high selectivity for HPO_4_^2−^ over HCO_3_^−^ was developed by Pierre and co-workers.^41^ Complexes [Eu-**4**]–[Eu-**13**] (Fig. 5) are based on tripodal ligand scaffold previously developed by the Raymond group.^42,43^ [Eu-**4**] was previously shown to bind weakly to oxalate, with no response to other anions despite possessing two open coordination sites. Pierre modified the ligand cap to favour formation of complexes with a nine-coordination geometry (*q* = 3) over eight. The backbone R substituent was varied to introduce groups capable of hydrogen bonding and increasing water solubility. Complexes [Eu-**5**], [Eu-**6**] and [Eu-**7**]^+^ were found to bind to HPO_4_^2−^, inducing a 36-fold, 20-fold and 5-fold increase in luminescence, respectively (Fig. 6a). The serine and glycine derived Eu(iii) complexes bound to HPO_4_^2−^ with 1 : 3 stoichiometries, with all three inner-sphere water molecules being displaced, whereas [Eu-**7**]^+^ bound to HPO_4_^2−^ in a 1 : 2 ratio, with one inner-sphere water remaining in the ternary adduct.

Selectivity for HPO_4_^2−^ was demonstrated through competitive binding studies with a range of anions, including HCO_3_^−^/CO_3_^2−^, F^−^, Cl^−^, SO_4_^2−^ and NO_3_^−^. Importantly, none of the complexes showed any affinity for HCO_3_^−^. Determination of stepwise association constants at pH 7.4 revealed that [Eu-**7**]^+^ displayed the highest affinity for HPO_4_^2−^ (log *K*^1^_a_ = 5.66; log *K*^2^_a_ = 5.67), followed by [Eu-**5**], which was attributed to stabilising hydrogen bonding interactions between HPO_4_^2−^ and the peripheral lysine N–H or serine O–H groups, respectively (Fig. 6b). Interestingly, the glutamatic and cysteic acid derived receptors, [Eu-**8**]^−^ and [Eu-**9**]^−^, showed no affinity for HPO_4_^2−^ or any other anion, highlighting the impact of introducing negatively charged groups peripherally to the open coordination site. Eu(iii) receptors of type [Eu-**4**]–[Eu-**13**] show great potential for the selective detection of phosphate in blood serum and demonstrate how selectivity can be fined tuned with the addition of appropriate pendant groups. The possible interaction with organic phosphate derivatives (*e.g.* AMP, ADP, ATP) could also be investigated.

In subsequent work, the potential relationship between the number of inner sphere water molecules and anion affinity was evaluated for a family of Eu(iii) complexes with different ligand cap sizes, forming eight- and nine-coordinate Eu(iii) complexes ([Eu-**4**], [Eu-**6**] [Eu-**10–13**], Fig. 5) with *q* = 2 or 3, respectively.^44^ Two out of the six complexes showed high affinity for HPO_4_^2−^ in water at pH 7.4, including [Eu-**10**] (log *K*^1^_a_ = 4.93; log *K*^2^_a_ = 5.46) and [Eu-**6**] (log *K*^1^_a_ = 3.67; log *K*^2^_a_ = 5.28; log *K*^3^_a_ = 5.56), with no observable binding to other anions including HCO_3_^−^ and acetate, although [Eu-**6**] showed affinity for cyanide above pH 10. Binding of HPO_4_^2−^ induced a gradual increase in Eu(iii) emission intensity at lower HPO_4_^2−^ concentrations, which accelerated at higher concentrations, consistent with a cooperative binding mechanism.

It was concluded that there was no correlation between the Eu(iii) complexes containing two or three inner-sphere water molecules and their affinity for HPO_4_^2−^. However, the observation of reversible CN^−^ binding to these complexes was investigated further, leading to the first Ln(iii)-based sensor for cyanide in water.^45^ Addition of CN^−^ to the lysine derivative [Eu-**7**]^+^ in water at pH 9.8, caused displacement of all three inner-sphere water molecules and a 9-fold enhancement in Eu(iii) emission intensity. Competitive binding of HPO_4_^2−^, CO_3_^2−^ and F^−^ was eliminated by the addition of excess Ca(ii) ions (forming insoluble salts), enabling selective sensing of CN^−^ in the range 0.1–1.5 mM.

**Fig. 7:**
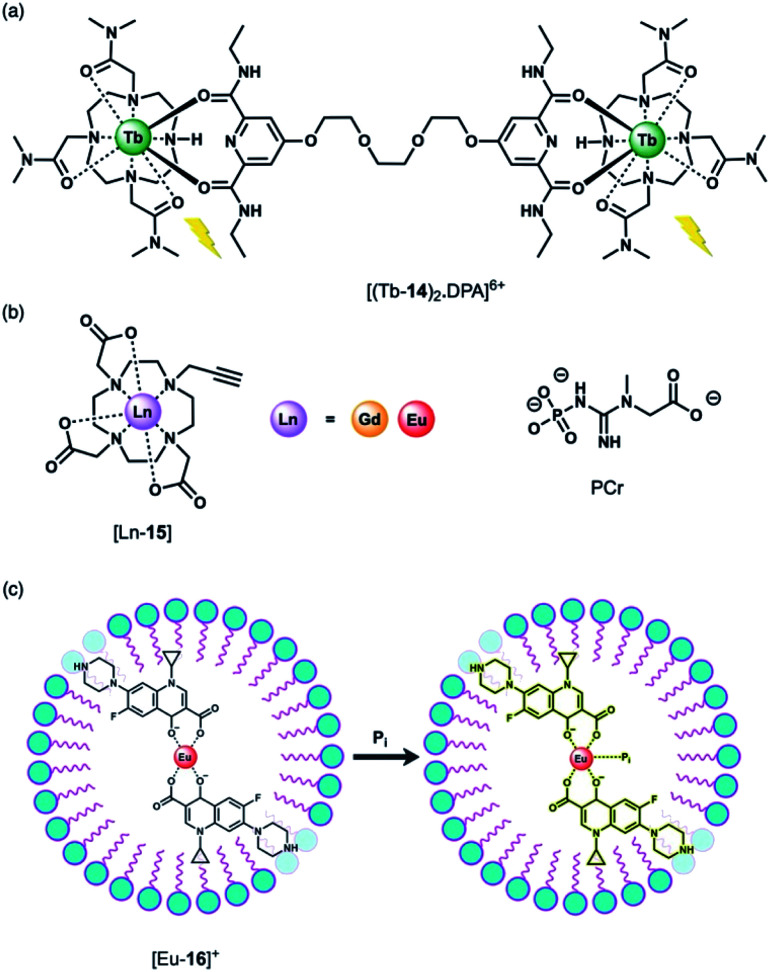
(a) Tb(iii)-Based displacement assay for HPO_4_^2−^ and NO_3_^−^; (b) alkyne-functionalised DO3A complex for assessing bulk phosphate species (PCr, HPO_4_^2−^); (c) schematic depiction of the detection of HPO_4_^2−^ (Pi) in water samples, using a Eu(iii)–CIP mixture in the presence of the surfactant SDBS.

Using a displacement assay, a Tb(iii)-based receptor with high selectivity for HPO_4_^2−^ and NO_3_^−^ was developed by Caffrey and Gunnlaugsson.^46^ The Tb(iii) complex [Tb-**14**]^3+^, based on a DTMA ligand, forms a ternary structure [(Tb-**14**)_2_·DPA]^6+^ upon the addition of a dipicolinic acid (DPA) derivative (Fig. 7a), which bridges two molecules of [Tb-**14**]^3+^. Subsequent addition of 1 equivalent of HPO_4_^2−^ to [(Tb-**14**)_2_·DPA]^6+^ in methanol disrupts the ternary structure, leading to 90% quenching of Tb(iii) luminescence, and 100% quenching following the addition of 2 equivalents of HPO_4_^2−^. Stern–Volmer constants for the stepwise displacement process were determined to be log *K*_a_ (1 : 1) = 6.6, and log *K*_a_ (2 : 1) = 11.9. Other anions including acetate and Cl^−^ induced only minor luminescence quenching (8% and 4%, respectively). On the other hand, NO_3_^−^ caused a larger 60% reduction in emission intensity (log *K*_a_ (1 : 1) = 4.7). Notably, this is the first time that NO_3_^−^ has been detected using a Ln(iii)-based displacement assay.

The binding of HPO_4_^2−^ to Ln(iii) complexes of a common macrocyclic ligand (Fig. 7b) has been studied by the Faulkner group using ^31^P NMR spectroscopy.^47^ Gd(iii) and Eu(iii) complexes of a DO3A ligand bearing a single alkyne group were prepared as precursors to more elaborate architectures. It was hypothesised that HPO_4_^2−^ would bind to the neutral complexes (*q* = 2) with low affinity and with relatively rapid exchange kinetics, enabling analysis of bulk phosphate species in solution. By increasing the concentration of the Gd(iii) complexes at pH 7.2 in the presence of HPO_4_^2−^, phosphocreatine (PCr) and ATP (8.7, 5.5 and 7.9 mM, respectively), ^31^P relaxation rates were determined, showing fast exchange and clear concentration dependent enhancement of the longitudinal ^31^P relaxation rates (similar to the way that conventional Gd(iii)-based MRI contrast agents enhance the relaxation of bulk water). The relaxivity (*r*_1_) values revealed the largest relaxation enhancement with phosphate, where *r*_1_ = 85.96 ± 1.00, compared with PCr (*r*_1_ = 54.97 ± 0.48) and ATP (*r*_1_ for α-P = 32.35 ± 0.46, β-P = 35.97 ± 0.35 and γ-P = 35.30 ± 0.64).

Anion titrations with the analogous Eu(iii) complex, [Eu-**15**] in 10 mM HEPES (pH 7.4) revealed modest increases in emission intensity and similar low affinity binding for phosphate, ATP and PCr (log *K*_a_ = 2.29 ± 0.03, 2.30 ± 0.02 and 2.08 ± 0.03, respectively), consistent with fast exchange. Lifetime measurements revealed that only one water molecule is displaced by HPO_4_^2−^, consistent with monodentate coordination of the anion. HPO_4_^2−^ binding to the Gd(iii) complex was thus expected to reduce water from the inner-sphere, which was confirmed by measurement of ^1^H water relaxation rates. The ^1^H relaxivity was found to be 16% lower in HPO_4_^2−^ solution compared with distilled water, reflecting the competition between water and phosphate for the Gd(iii) centre, which reduces the concentration of bound water molecules relative to the bulk. This study highlights the potential for ratiometric determination of extracellular HPO_4_^2−^ using multinuclear (^31^P and ^1^H) relaxometric measurements, although the authors acknowledge that dual measurements would require advances in current instrumentation.

Wu and Tong developed a “turn-on” fluorescent system for the selective detection of HPO_4_^2−^ in environmental water samples (Fig. 7c).^48^ The multicomponent system is based on Eu(iii) coordination of ciprofloxacin (CIP), a non-toxic fluoroquinoline antibiotic. The fluorescence of CIP is quenched completely when coordinated to Eu(iii) in the presence of the surfactant sodium dodecylbenzenesulfonate (SDBS). Upon addition of HPO_4_^2−^ to [Eu-**16**]^+^, the blue fluorescence of CIP is recovered. HPO_4_^2−^ is proposed to bind to the Eu(iii) ion, weakening the energy transfer from the CIP ligand to the Eu(iii) metal, although the complete dissociation of CIP from Eu(iii) is also possible. This sensing system was evaluated in environmental water samples (including wastewater and surface water), demonstrating the detection of HPO_4_^2−^ within the concentration range 0.02–4.0 μM. The selectivity was examined and a slight interference with Cr(iii) ions was observed, whereas no other cations or anions tested induced a significant response.

## Receptors for nucleoside phosphate anions

Nucleoside phosphate (NP) anions such as ATP, GTP, ADP, UDP, AMP are involved in a wide range of biological processes, including energy transfer, DNA synthesis, intracellular signalling and regulation of enzyme activity.^49^ ATP is the most abundant NP anion in cells and is the primary chemical energy source in living systems. The majority of ATP is generated in the mitochondria by oxidative phosphorylation, and serves as a substrate for several enzymes, including kinases, ATPases and RNA polymerases. The concentration of ATP varies significantly from nanomolar extracellular levels to millimolar levels (1–5 mM) in certain cellular organelles.^50–52^ In comparison, ADP is present in much lower levels inside cells (50–200 μM), with the ATP/ADP ratio ranging between 5–100.^53^

### ATP and ADP receptors

The development of receptors that can signal ATP levels *in cellulo* could provide a better understanding of the way in which energy is produced, transported and consumed within the cell.^54,55^ Moreover, a receptor that can discriminate between ATP and ADP could be used for monitoring of kinase enzyme activity in real-time.^56^ Kinases catalyse the phosphorylation of proteins (converting ATP to ADP in the process), and constitute one of the most promising drug targets for the treatment of cancer.^7^ An ATP or ADP-selective receptor could facilitate high throughput screening campaigns for the identification of potent kinase inhibitors,^7,8^ surmounting the limitations of current bioassay technologies that rely upon unstable antibodies, or radioactive substrates. To be effective in such applications, the receptor should exhibit rapid and reversible anion binding to prevent perturbation of enzyme reaction rates, and operate in the presence of Mg(ii) and Ca(ii) ions, which compete for ATP (and ADP) binding.^57^

**Fig. 8:**
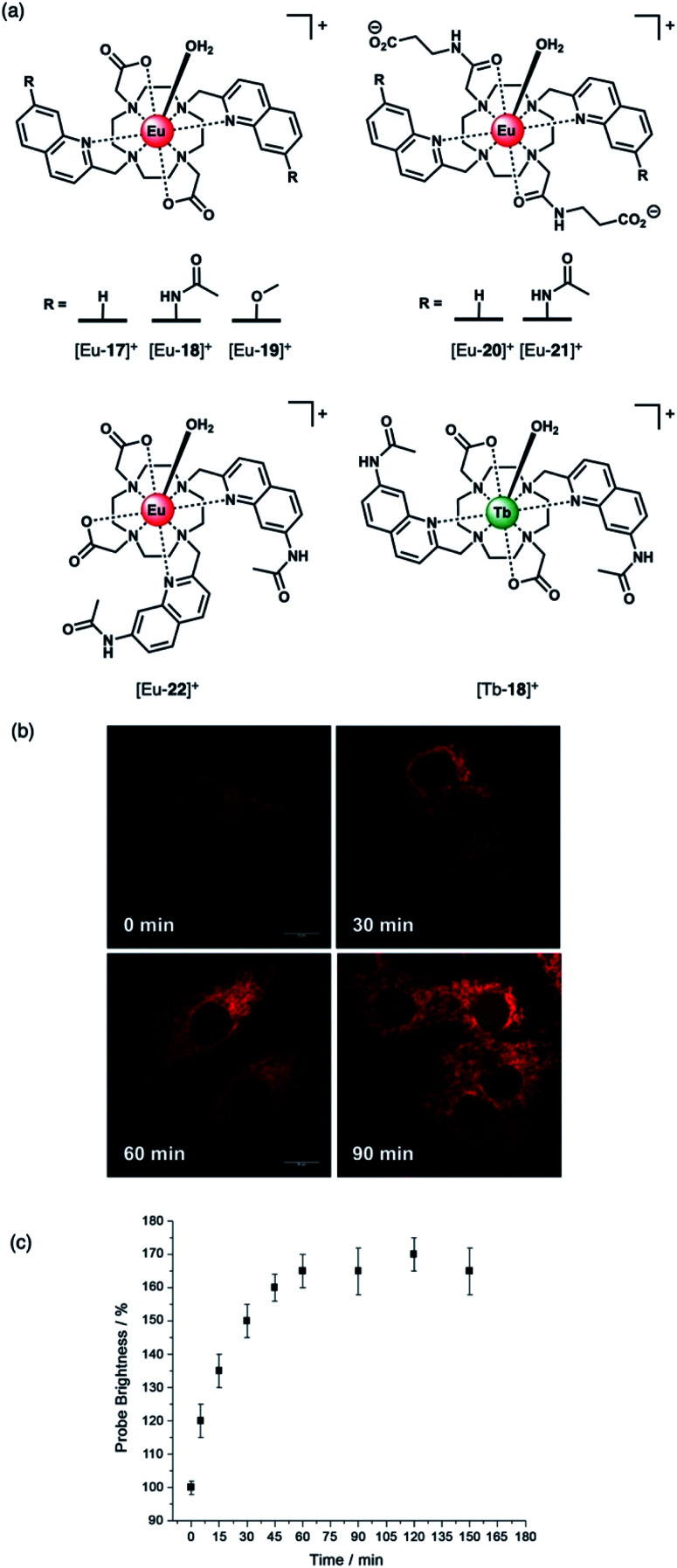
(a) Family of cationic Eu(ii) and Tb(iii) complexes capable of discriminating between nucleoside tri-, di- and monophosphate anions (*e.g.* ATP, ADP and AMP); (b) Real-time monitoring of elevated ATP levels in the mitochondria of living cells. Time-lapsed LSCM images of NIH-3T3 cells stained with [Eu-**21**]^+^ (50 μM, *λ*_exc_ 355 nm, *λ*_em_ 605–720 nm) before (0 min) and after treatment with staurosporine (10 nM), a potent inhibitor of protein kinases; (c) time-dependent increase in emission intensity (605–720 nm) of cells stained with [Eu-**21**]^+^ following treatment with staurosporine (10 nM). Reproduced from ref. ^58^ with permission from Wiley-VCH, copyright 2018.

Butler and co-workers synthesised a series of cationic *C*_2_-symmetric Eu(iii) complexes [Eu-**17**]^+^–[Eu-**21**]^+^ (Fig. 8a),^58^ and evaluated their binding towards NP anions at physiological pH. Complexes [Eu-**18**]^+^ and [Eu-**21**]^+^ showed excellent discrimination between ATP, ADP and AMP in 10 mM HEPES buffer in the presence of 5 mM Mg(ii) ions. Anion binding occurs directly at the metal centre with displacement of the inner-sphere water molecule, increasing the Eu(iii) emission intensity and lifetime. Complex [Eu-**21**]^+^ binds most strongly to ATP (log *K*_a_ = 5.8 in 10 mM HEPES, pH 7.0), resulting in a 24-fold increase in intensity of the Δ*J* = 2 emission band, whereas ADP causes a smaller increase in emission, despite showing similar affinity. Binding of ATP was approximately 10 times stronger than AMP or pyrophosphate (P_2_O_7_^4−^), and 100 times stronger than HPO_4_^2−^ and HCO_3_^−^ (log *K*_a_ = 2.7 and 3.0, respectively). The high affinity of [Eu-**21**]^+^ for ATP is attributed primarily to strong metal-ligand interactions, strengthened by hydrogen bonding to the quinoline amide arms projecting from the same face of the receptor. Solution NMR analysis, supported by DFT calculations and X-ray crystallography, indicate a bidentate binding mode of ATP to the Eu(iii) complexes, *via* the α- and γ-phosphate groups, forming a 1 : 1 host–guest complex in aqueous solution with fast binding kinetics on the NMR timescale.^59^

The distinctive luminescence response of [Eu-**21**]^+^ for ATP enabled its detection in a simulated biological medium containing NaCl, Mg^2+^, ADP, GTP, UTP and human serum albumin.^58^ The increase in intensity of the ▵*J* = 2 band was approximately linear over the biologically relevant ATP range of 0.3–8.0 mM. [Eu-**21**]^+^ was found to permeate mammalian (NIH-3T3) cells and localise to the mitochondria selectively, permitting real-time visualization of elevated mitochondrial ATP levels, following treatment with a broad spectrum kinase inhibitor, staurosporine (Fig. 8b). Additionally, it was possible to image depleted ATP levels upon treatment with potassium cyanide (an inhibitor of oxidative phosphorylation), under glucose starvation conditions.

The structurally related complex [Eu-**18**]^+^ showed lower affinity for both ATP and ADP (log *K*_a_ = 4.4 and log *K*_a_ = 4.6, respectively) compared with [Eu-**21**]^+^, but gave a much larger increase in emission for ADP in the presence of Mg(ii) ions.^60^ The weaker binding of [Eu-**18**]^+^ to ATP (and ADP) is ascribed to the decrease in the electropositive nature of the metal ion due to the presence of two pendant carboxylate donors, compared with the neutral carbonyl amide donors in [Eu-**21**]^+^. In the presence of Mg(ii) ions, the interaction of [Eu-**18**]^+^ with ATP is significantly reduced, due to competitive Mg–ATP binding, enabling a selective response for ADP to be attained. The ability of [Eu-**18**]^+^ to signal ADP in the presence of ATP was utilised to monitor a phosphorylation reaction catalysed by protein kinase A, by following the intensity ratio at 616/600 nm as a function of the increasing ratio of ADP/ATP.^60^

**Fig. 9:**
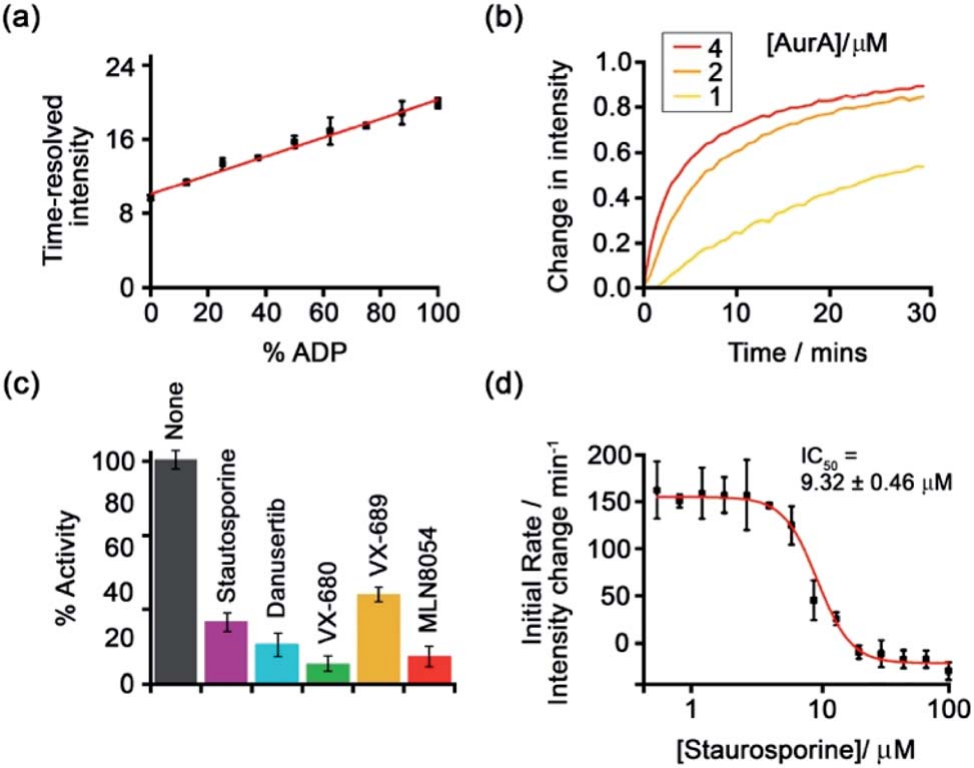
Microplate-based assay for real-time monitoring of kinase activity using [Eu-**18**]^+^. (a) Kinase simulation in standard assay conditions (total ATP + ADP = 1 mM, 5 mM MgCl_2_, 8 μM [Eu-**18**]^+^, 10 mM HEPES, pH 7.0), measuring the time-resolved luminescence intensity of differing ratios of ADP/ATP; (b) real-time monitoring of Aurora A kinase reactions at different concentrations of enzyme; (c) inhibition of Aurora A (1 μM) by a range of known inhibitors measured in real-time; (d) IC_50_ derived from the luminescent titration of staurosporine into Aurora A (50 nM). Reproduced (adapted) from ref. ^6^ with permission from the Royal Society of Chemistry, copyright 2019.

Subsequently, [Eu-**18**]^+^ was developed into a miniaturised assay for real-time monitoring of a variety of pharmaceutically important enzymes that generate NP anions, including kinases (ATP into ADP, Fig. 9), glycosyltransferases (UDP-sugar into UDP), and phosphodiesterases (cAMP into AMP).^6^ In each case, changes in the NP product/substrate ratio are monitored by time-resolved luminescence using a standard plate reader, allowing different enzyme classes to monitored by a convenient increase-in-signal format. Unlike the majority of commercial enzyme assays, [Eu-**18**]^+^ can operate at physiological concentrations of NP anions (*e.g.* 1–5 mM ATP for kinase reactions), permitting screening of inhibitors of low activity enzymes. The impact of a range of known inhibitors on the activity of Aurora A kinase was assessed using [Eu-**18**]^+^ (Fig. 9c), and an inhibitor titration was conducted for the inhibitor staurosporine (Fig. 9d), giving an IC_50_ of 9.32 ± 0.46 mM, comparable with previously reported values.

**Fig. 10:**
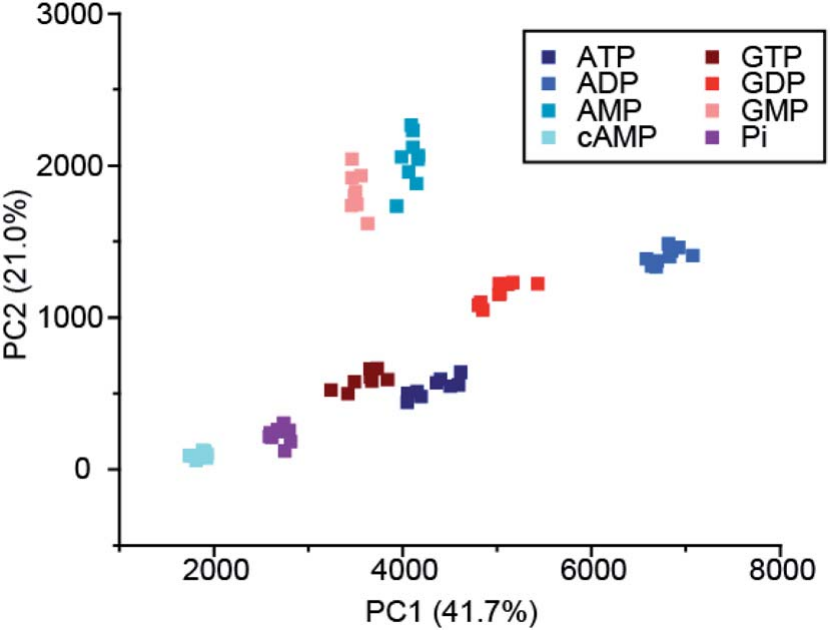
Principle component analysis (PCA) score plots of % change in emission intensity of [Eu-**18**]^+^ (8 μM), [Eu-**21**]^+^ (13 μM), [Eu-**19**]^+^ (10 μM) and [Tb-**18**]^+^ (15 μM) with the adenosine and guanosine series of nucleoside polyphosphate anions (1 mM), in 10 mM HEPES, pH 7.0. Reproduced (adapted) from ref. ^59^ with permission from the Royal Society of Chemistry, copyright 2020.

Recently, Butler and co-workers extended the library of cationic Ln(iii) receptors and established how their phosphoanion binding properties can be tuned through modifications in the ligand structure.^59^ The relative positions of the pendant quinoline arms on the macrocyclic ligand were shown to significantly impact on host-anion affinity and stability, with the *trans*-related quinoline groups of [Ln-**18**]^+^, [Eu-**19**]^+^ and [Eu-**21**]^+^, providing sufficient flexibility to accommodate ATP (and ADP), whereas the *cis*-orientation of [Eu-**22**]^+^ reduces stability of the host-anion complex, leading to metal ion dissociation over time. Anion affinity can be increased by an order of magnitude by incorporating H-bond donors in the quinoline arms, or by introducing neutral pendant donors to increase the local positive charge at the Ln(iii) centre. Using a combination of four of the Eu/Tb receptors ([Eu-**18**]^+^, [Tb-**18**]^+^, [Eu-**19**]^+^ and [Eu-**21**]^+^) it was possible to discriminate eight NP anions (ATP, ADP, AMP, GTP, GD(iii)P, GMP, cAMP, Pi) in a sensing array, using principle component analysis (Fig. 10). The sensing array takes advantage of the differential emission intensities and lifetimes of the four Ln(iii) receptors to generate additional anion selectivity through time-resolved measurements.

**Fig. 11:**
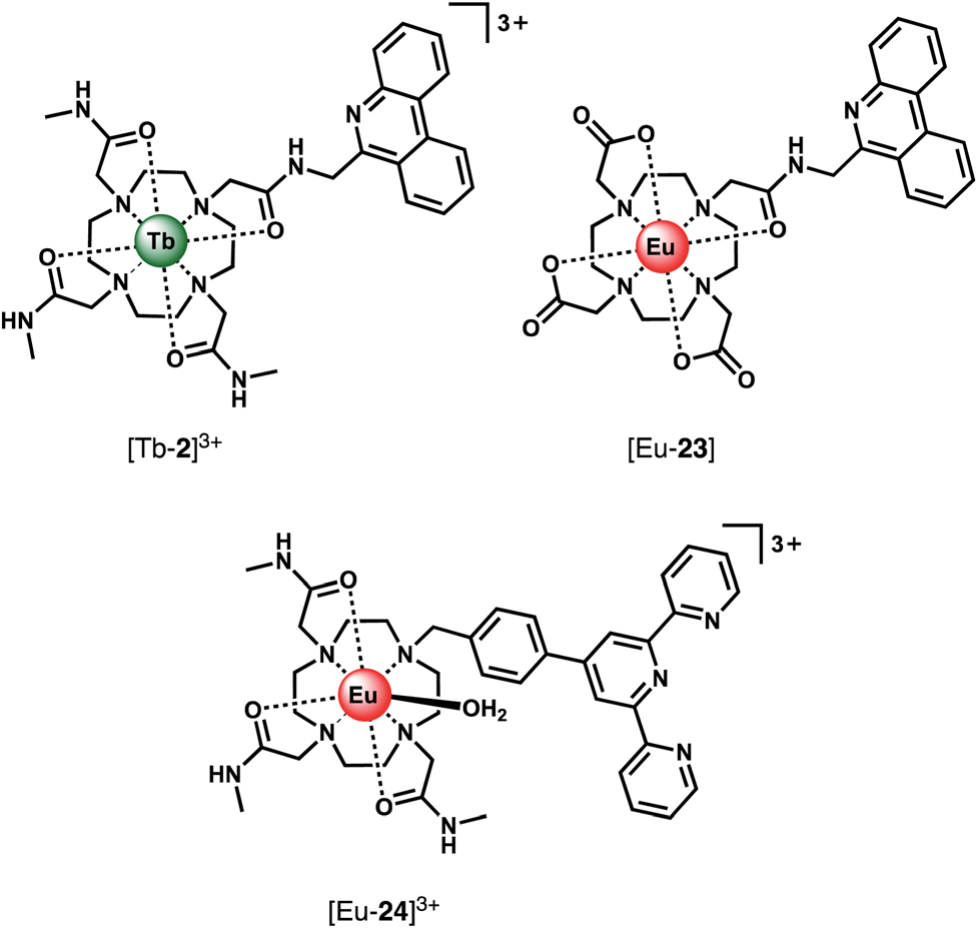
Eu(iii) and Tb(iii) complexes capable of selective binding ATP (and GTP) through π–π stacking and electrostatic interactions, inducing quenching of luminescence.

**Fig. 12:**
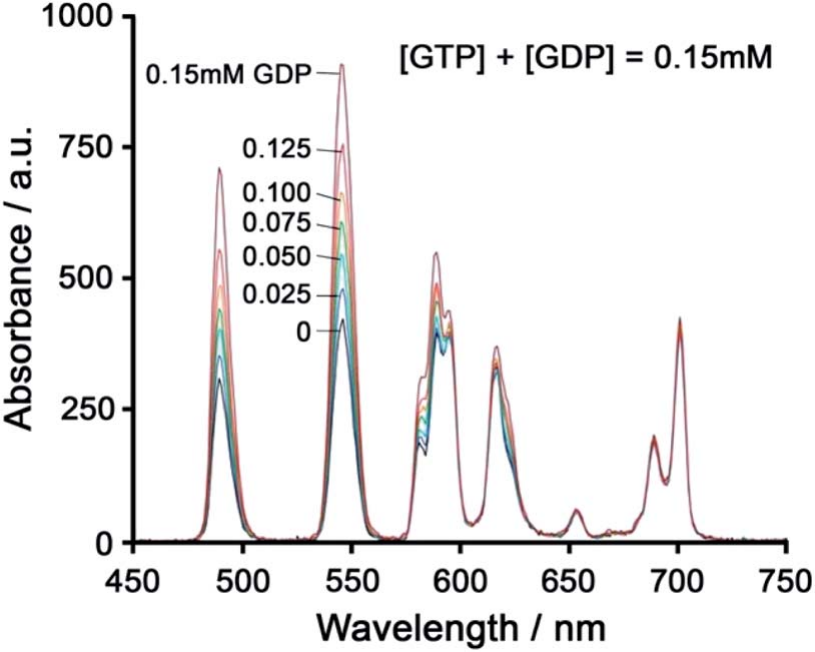
Ratiometric determination of GTP/GDP ratio using time-delayed luminescence of a mixture of [Tb-**2**]^3+^ and [Eu-**23**]. Reproduced from ref. ^61^ with permission from the Royal Society of Chemistry, copyright 2013.

Using a different approach, Pierre and co-workers prepared a Tb(iii) receptor [Tb-**2**]^3+^ (Fig. 11) capable of binding ATP through π–π stacking between the adenine moiety and the phenanthridine antenna.^36^ The positively charged Tb(iii) complex engages in electrostatic interactions with ATP, which are stronger than for ADP and AMP. The π–π stacking interaction promotes photoinduced electron transfer (PET), which prevents energy transfer from the antenna to the Tb(iii) centre, thus quenching luminescence. Similar quenching was observed in the presence of GTP, whereas pyrimidine nucleosides (UTP, CTP) did not cause PET quenching. Notably, there is no direct coordination of phosphate groups to the Tb(iii) ion, hence the overall affinity for ATP and GTP is relatively low. Stern–Volmer plots conducted in 10 mM Tris buffer (pH 7) revealed high selectivity towards ATP over ADP/AMP (and similarly, for GTP over GDP/GMP). Subsequently, [Tb-**2**]^3+^ was combined with a non-responsive neutral Eu(iii) complex [Eu-**23**] to produce a ratiometric cocktail for monitoring ATP/ADP or GTP/GDP ratios, by following the change in Tb/Eu emission ratio at 545/700 nm (Fig. 12).^61^ This mixture of Eu(iii)/Tb(iii) complexes could potentially be used for monitoring GTPase activity in real-time.

Using a very similar design, Tang *et al.* developed a Eu(iii) complex, [Eu-**24**]^3+^, with an appended terpyridine antenna for ATP sensing (Fig. 11).^62^ The proposed binding mode involves an initial 2 : 1 host–guest complex when 0.5 equivalents of ATP is added, wherein ATP stacks between the terpyridine units of two Eu(iii) complexes, causing an 8.3-fold increase in emission. Further equivalents of ATP cause a loss of stacking of the sandwich-type structure, resulting in a 1 : 1 complex and associated decrease in emission intensity. Anion selectivity studies were conducted in water at pH 6.8 (due to the emission intensity being sensitive to pH changes in the range 6.8–8.0), revealing that other phosphate anions and HCO_3_^−^ have small influences on the emission, while citrate notably impacts the selectivity of the receptor for ATP. Although this complex can distinguish ATP from ADP/AMP in aqueous solution, its use in biological systems would unfortunately be problematic due to its pH sensitivity and the specific ratio of host : guest required to ‘turn-on’ emission. In the presence of more than 0.5 equivalents of ATP, the emission will be quenched due to a 1 : 1 binding mode being preferential.

**Fig. 13:**
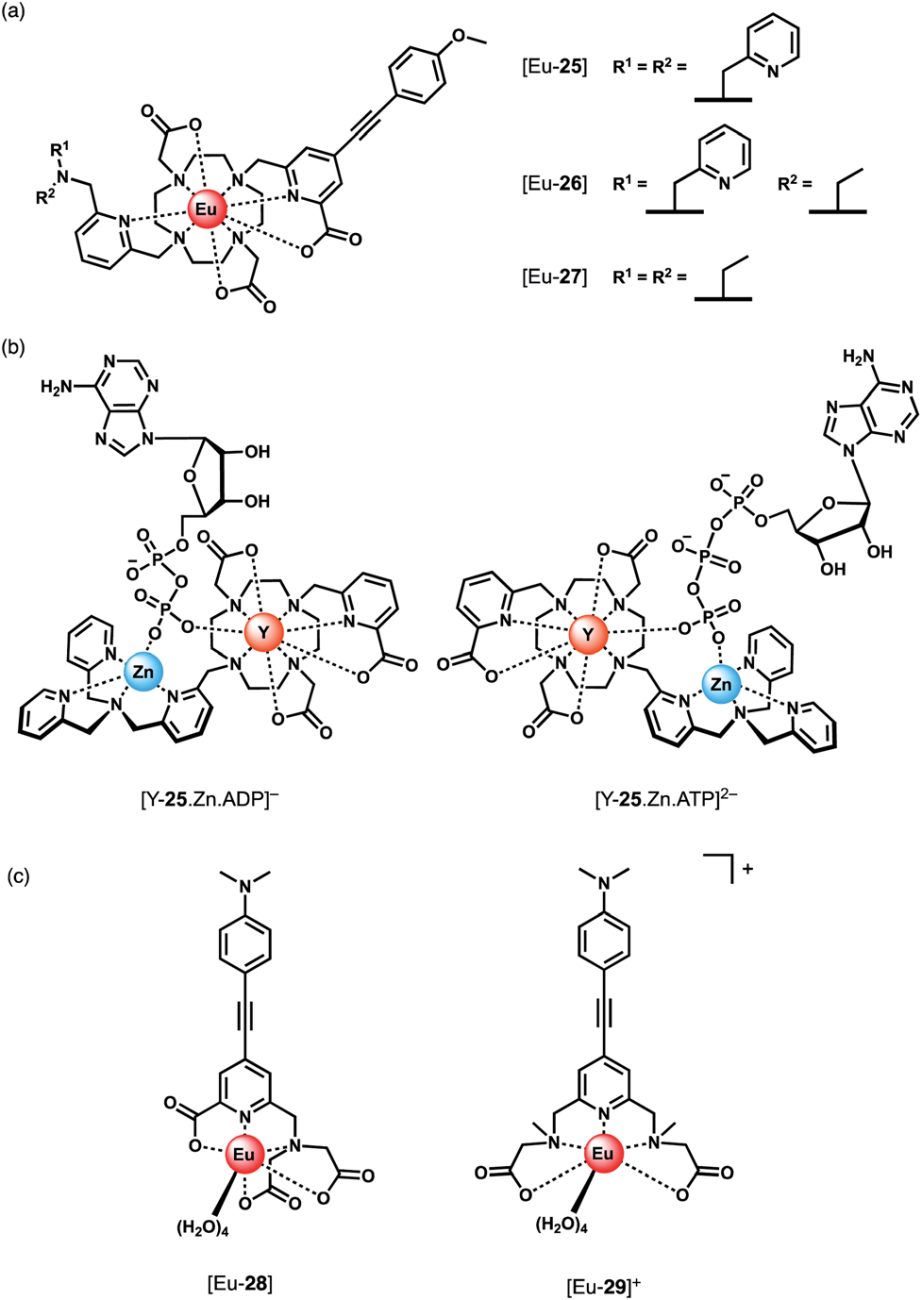
(a) Parker's CPL probes for the discrimination of ATP and ADP. (b) Structures showing differing chirality of the ATP and ADP adducts of complex [Y-**25**·Zn]^2+^. Optimised DFT geometries indicate the Δ diastereomer is preferred for ATP and the Λ isomer for ADP. (c) Pentadentate Eu(iii) complexes developed by Schäferling for ATP detection.

Parker and co-workers devised a short series of Eu(iii) complexes, capable of monitoring the ATP/ADP ratio by induced circularly polarised luminescence (CPL).^63^ Complexes [Eu-**27**]–[Eu-**29**] (Fig. 13) are based on the strongly emissive Eurotracker® series of probes,^64,65^ containing one alkynyl-pyridine chromophore and a substituted picolylamine moiety, containing either two picolyl groups, two ethyl groups, or one of each. The differences in the ligand structure were found to modulate binding affinities for both Zn(ii) and NP anions. An increase in emission intensity of [Eu-**25**] and [Eu-**26**] is observed when Zn(ii) is bound, followed by amplification of the signal when the NP anion is bound. Replacement of the picolyl moieties with an ethyl group caused a decrease in binding affinities for NP anions (AMP, ADP, ATP), from [Eu-**25**] (log *K*_a_ = 5.6–6.3) to [Eu-**26**] (log *K*_a_ = 3.3–4.6) to [Eu-**27**] (very weak binding indicated by anion titrations not reaching saturation, log *K*_a_ values not reported). Interestingly, the affinity of the binuclear complex [Eu-**25**·Zn]^2+^ for AMP, ADP and ATP is lower (log *K*_a_ = 4.5–4.8) than for [Eu-**25**] in the absence of Zn(ii), suggesting that the protonated tertiary amine of [Eu-**25**] provides significant stabilisation of the bound NP anion, through ionic hydrogen bonding interactions.

Most notably, the binding of ATP and ADP to the binuclear complex [Eu-**25**·Zn]^2+^ in water (0.1 M HEPES, pH 7.4) induced strong CPL signals of opposite sign, which enabled monitoring of changes in the emission dissymmetry factor, *g*_em_, as a function of the ratio of ATP/ADP. CPL spectral data supported by DFT calculations revealed differences in the chirality of the ATP and ADP adducts of the [Y-**25**·Zn]^2+^ complex, which differ in the arrangement of the exocyclic ring substituents (Λ/Δ). The optimised DFT geometries of the adducts indicate that the terminal phosphate of the nucleotide bridges the Zn(ii) and Eu(iii) ions, and suggest the Δ diastereomer is preferred for ATP and the Λ isomer for ADP or AMP (Fig. 13).

Schäferling and co-workers prepared a series of multidentate Eu(iii) complexes of varying stability (7-coordinate down to 3-coordinate complexes), each containing an alkynyl pyridine group for sensitisation of Eu(iii) emission.^66,67^ The neutral pentadentate Eu(iii) complex [Eu-**28**] was found to have four coordinated water molecules, and showed a 60-fold increase in Eu(iii) emission intensity upon the addition of 1 mM ATP (or 1 mM pyrophosphate), *versus* a 27-fold increase with ADP. However, in the presence of 5 mM Mg^2+^ ions, discrimination between ATP and ADP was lost, due to the competitive interaction of ATP with Mg^2+^. However, at lower concentrations of Mg^2+^ (0.05 mM), it was possible to track the ATPase catalysed conversion of ATP to ADP, by measuring a decrease in luminescence as a function of time.

Subsequently, the same authors showed that the related cationic Eu(iii) complex [Eu-**29**]^+^, bearing two ethyl 2-(methylamino) acetate arms, gave a large increase in luminescence for ATP, compared with other NP anions (ADP, CTP). The emission intensity increased linearly over the ATP concentration range 0–20 μM. A 1 : 1 binding mode was assumed although no binding constants were reported. [Eu-**29**]^+^ displayed significant ligand fluorescence centred at 490 nm, indicating less efficient energy transfer from the ligand to the Eu(iii) centre compared with [Eu-**28**], potentially arising from weaker bonds between the Eu(iii) ion and the ethyl 2-(methylamino) arms. Despite this, the high sensitivity of [Eu-**29**]^+^ towards ATP was utilised to monitor apyrase activity at pH 6.5, again by following a decrease in Eu(iii) luminescence as ATP is converted to ADP/AMP.

### Nucleoside monophosphate receptors

**Fig. 14:**
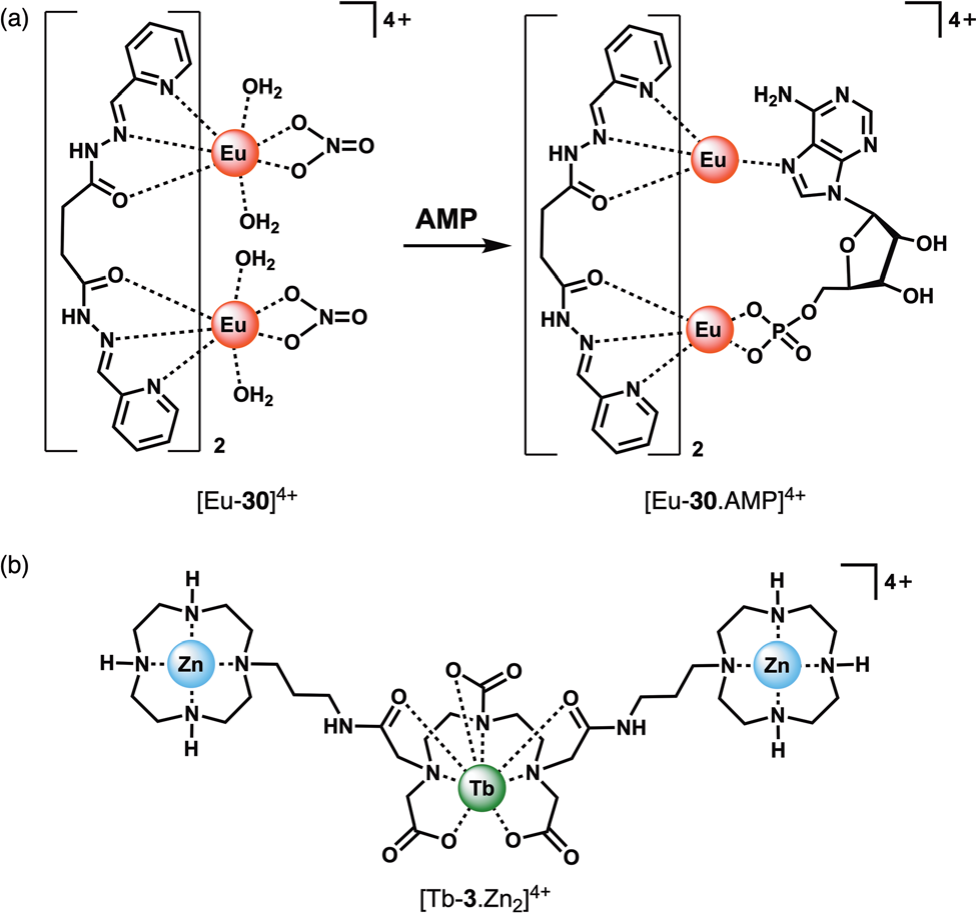
Ln(iii) receptors for the selective recognition of nucleoside monophosphate anions. (a) Proposed binding mode of AMP to Eu(iii) helicate [Eu-**30**]^4+^; (b) trinuclear complex [Tb-**3**·Zn_2_]^4+^ capable of signalling GMP.

The majority of NP receptors reported to date have targeted ATP or ADP. In comparison, receptors capable of selectively binding the monophosphate anion AMP are rare. This requires high geometric complementarity between the receptor and AMP to outweigh the coulombic attraction for the more negatively charged anions, ATP and ADP. Albrecht and co-workers developed a luminescent Eu(iii) helicate, [Eu-**30**]^4+^, that exhibits unique selectivity for AMP (Fig. 14a).^68^ The molecule comprises an unsaturated double-stranded dinuclear helicate formed from two bis(tridentate) ligands and two Eu(iii) centres. A wide range of anionic co-ligands for the helicate were screened in 10 mM HEPES (pH 7.4), with only AMP binding causing an enhancement in luminescence (*Φ* increases from 1.8 to 6.1%). A binding constant for AMP of log *K*_a_ = 3.83 ± 0.01 was determined. AMP appears to be the perfect size and shape match to bridge the two Eu(iii) centres, displacing the quenching solvent and counterions in the process. The proposed binding mode involves phosphate coordination to one the Eu(iii) centres, and subsequent binding of an adenine nitrogen atom to the second metal centre, forming a triple-stranded helical structure. The remarkable discrimination of AMP from ADP and ATP, combined with the sensitive detection limit of 2 μM, indicates that [Eu-**30**]^4+^ could be used for detecting AMP in biological media, although the stability of the Eu(iii) helicate in the presence of proteins (which could potentially cause metal ion dissociation) would need to be investigated.

Guanosine monophosphate (GMP) is an important cell signalling molecule produced from cyclic guanosine monophosphate (cGMP), a reaction catalysed by phosphodiesterases (PDEs).^69^ Receptors capable of detecting GMP could enable processes mediated by PDEs to be monitored in real-time.^6^ The selective detection of GMP in aqueous solution was accomplished with a Tb(iii)–bisZn(ii) complex, [Tb-**3**·Zn_2_]^4+^, developed by Tuck and co-workers (Fig. 14b).^37^ The Tb(iii) centre can be sensitised by proximal guanine nucleotides: the addition of GMP to [Tb-**3**·Zn_2_]^4+^ in 10 mM HEPES buffer (pH 7.4) induced an 87-fold enhancement in Tb(iii) emission intensity, whereas GDP (0.5 equiv.) and GTP (0.25 equiv.) induced a 28-fold and 12-fold increase in luminescence respectively, with further equivalents of anions causing a decrease in luminescence. The presence of Zn(ii) ions in the receptor is important for GMP recognition, since addition of one equivalent GMP to [Tb-**3**] alone (in the absence of Zn(ii) ions) results in a much smaller 2.5-fold increase in Tb(iii) luminescence. Analysis of the titration data indicated that GMP binds to a single Zn(ii) ion forming a 1 : 1 complex (Fig. 4d), whereas di- and triphosphates initially appear to bridge two host molecules in a 2 : 1 binding mode. The stronger luminescence of the GMP adduct compared with those of GTP and GDP is attributed to the closer proximity of the guanine group of GMP to the Tb(iii) centre, permitting more efficient sensitisation by Förster resonance energy transfer.

Competition studies revealed that GMP binds preferentially to [Tb-**3**·Zn_2_]^4+^ over AMP and CMP; however, UMP has a stronger binding affinity and is able to displace GMP causing a decrease in luminescence. The Tb(iii) complex binds acyclic nucleotides (*e.g.* GMP) more strongly than the cyclic counter parts (*e.g.* cGMP), owing to the increased negative charge of the former species. The potential biological utility of this receptor was demonstrated by monitoring the PDE catalysed conversion of cGMP to GMP, by following the increase in Tb(iii) emission at 544 nm. The Michaelis Menten constant was estimated to be *K*_M_ = 3.63 ± 0.33 mM, consistent with the literature value.

### Receptors for chiral phosphoanions

**Fig. 15:**
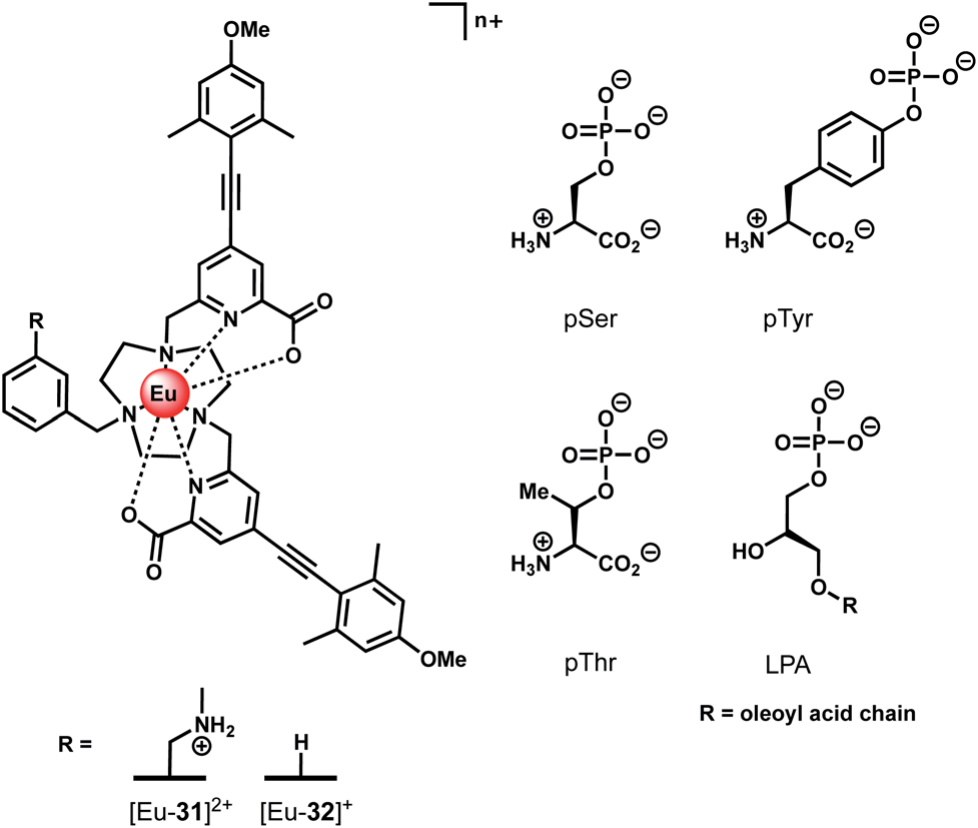
Structures of emissive Eu(iii) complexes based on TACN, which bind and signal chiral phosphorylated amino acids and LPA, using CPL spectroscopy.

Ln(iii) complexes have been studied extensively as circularly polarised luminescence (CPL) probes by the Parker group.^70,71^ When the environment around a Ln(iii) ion is chiral, the metal may display highly polarised emission, measured in terms of the dissymmetry factor, or *g*_em_ value. A strong CPL signal may be induced upon binding of chiral anions or biomolecules, offering exciting potential for biological imaging of chiral species.^71^ Two Eu(iii) complexes based on triazacyclononane (TACN) containing two strongly absorbing pyridylalkynylaryl antennae (Fig. 15) were recently synthesised, which show selective CPL signalling of chiral phosphoanions, including phosphoserine (pSer), phosphothreonine (pThr) and lysophosphatidic acid (LPA).^72^ The ligands were designed to disfavour chelating anions with small bite angles, such as HCO_3_^−^, by incorporating a benzyl group to impose steric demand around the Ln(iii) ion, which additionally resulted in no inner-sphere water molecules.

Anion binding was examined in a 1 : 1 water/methanol mixture (buffered with 10 mM HEPES) due to the complexes not being fully soluble in water. Both Eu(iii) complexes show reversible binding of lactate (log *K*_a_ = 4.37 ± 0.07 and 3.15 ± 0.07 for [Eu-**31**]^2+^ and [Eu-**32**]^+^, respectively), however neither complex bound HCO_3_^−^. Phosphate showed an interaction with [Eu-**31**]^+^ (log *K*_a_ = 4.2 ± 0.1), whereas no change in emission was observed for [Eu-**32**]^+^. Addition of chiral phosphorylated amino acids, pSer, pThr and pTyr, resulted in subtle changes in the emission spectra but induced strong CPL signals for pSer and pThr, whereas no induced CPL signal was observed for pTyr. The lack of induced CPL activity with pTyr is attributed to the chiral centre being more remote compared with the pSer or pThr adducts. The emission dissymmetry values, *g*_em_, were determined for pThr and pSer to be +0.08 and +0.04 (at 593 nm), respectively. The binding affinity of [Eu-**31**]^2+^ for pSer, pThr and pTyr was found to be the same (log *K*_a_ = 4.80), revealing no preference for a particular phosphorylated amino acid.

## Receptors for bicarbonate

Bicarbonate (HCO_3_^−^) plays important roles in the regulation of cellular pH, removal of metabolic waste and kidney function.^73^ Several diseases are associated with misregulation of HCO_3_^−^ levels due to mutations in HCO_3_^−^ transporting proteins, including renal diseases, haemolytic anaemia and glaucoma.^5^ HCO_3_^−^ is therefore an important target for receptor development. Probes capable of monitoring spatio-temporal HCO_3_^−^ dynamics in living cells would provide a deeper understanding of the diverse biological processes this anion controls, potentially facilitating the development of new therapeutic agents (*e.g.* channel replacement therapies).

**Fig. 16:**
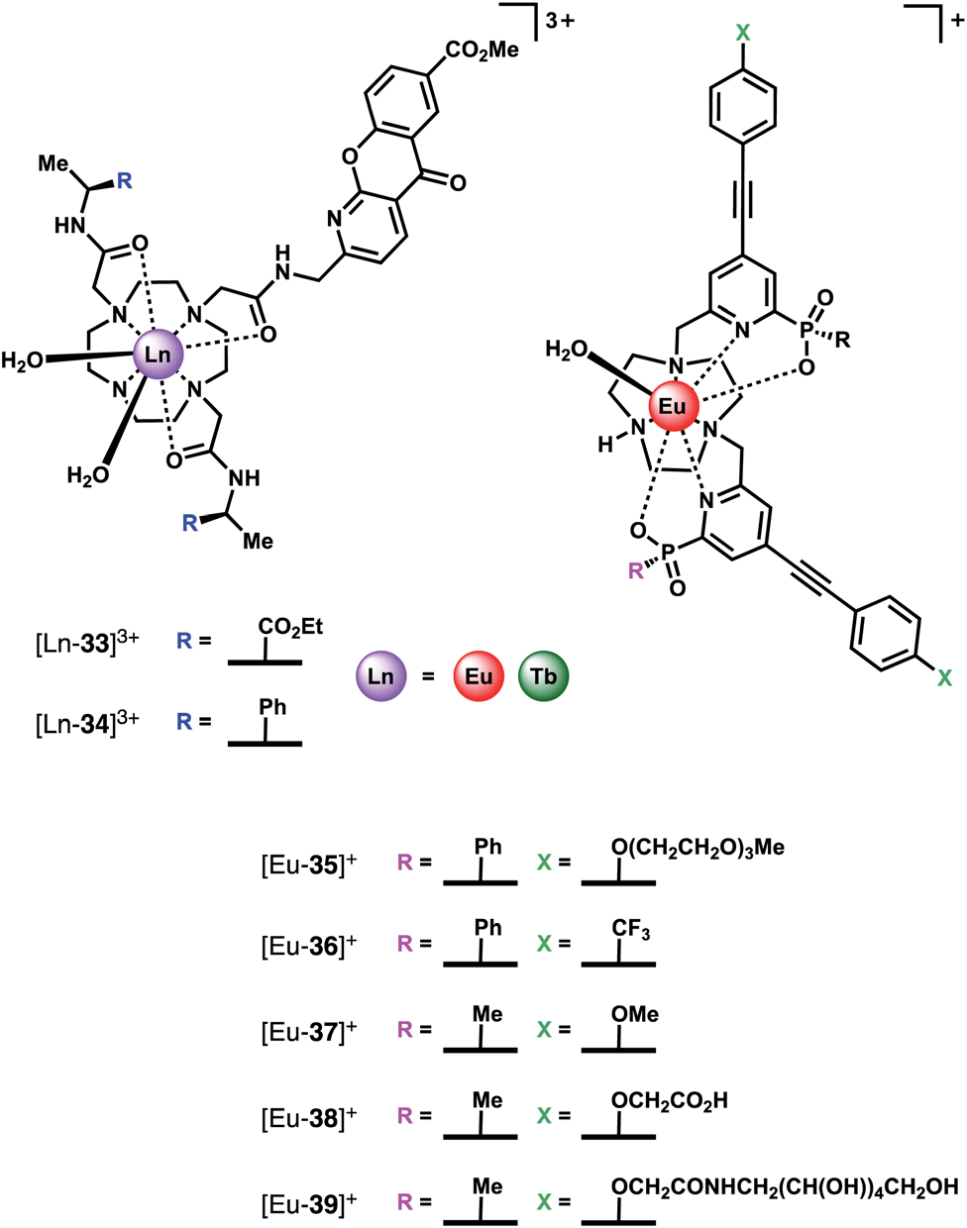
Structures of HCO_3_^−^ selective Ln(iii) receptors, utilised for reporting HCO_3_^−^ levels in human serum and in living cells.

The selective binding of HCO_3_^−^ to tricationic complexes [Ln-**33**]^3+^ and [Ln-**34**]^3+^ (Ln = Eu, Tb) (Fig. 16) bearing an amide-linked azaxanthone sensitiser was examined by the Parker group.^4,34^ The Eu(iii) complexes bind reversibly to HCO_3_^−^ in water (log *K*_a_ = 3.85, 0.1 M NaCl, pH 7.4), causing displacement of the two inner sphere water molecules and a large increase in emission intensity, particularly the ▵*J* = 2 band. Notably, the Tb(iii) analogue of [Eu-**33**]^3+^ showed much smaller changes in emission intensity with added HCO_3_^−^, although binding still occurred (log *K*_a_ = 3.00). A bridging binding mode of the carbonate anion (CO_3_^2−^) was proposed, consistent with previous NMR, mass spectrometric and X-ray studies.^30,74^ Binding interactions with other anions including HPO_4_^2−^, lactate, citrate and human serum albumin were determined, with citrate showing the strongest binding (log *K*_a_ = 4.80). Citrate was also the only anion that induced a similar change in emission spectrum to HCO_3_^−^. Considering that the intracellular concentration of HCO_3_^−^ (10–12 mM) is significantly higher than citrate (2–4 mM) it was hypothesised that HCO_3_^−^ would effectively compete with citrate for interaction with the receptor in a cellular environment.

**Fig. 17:**
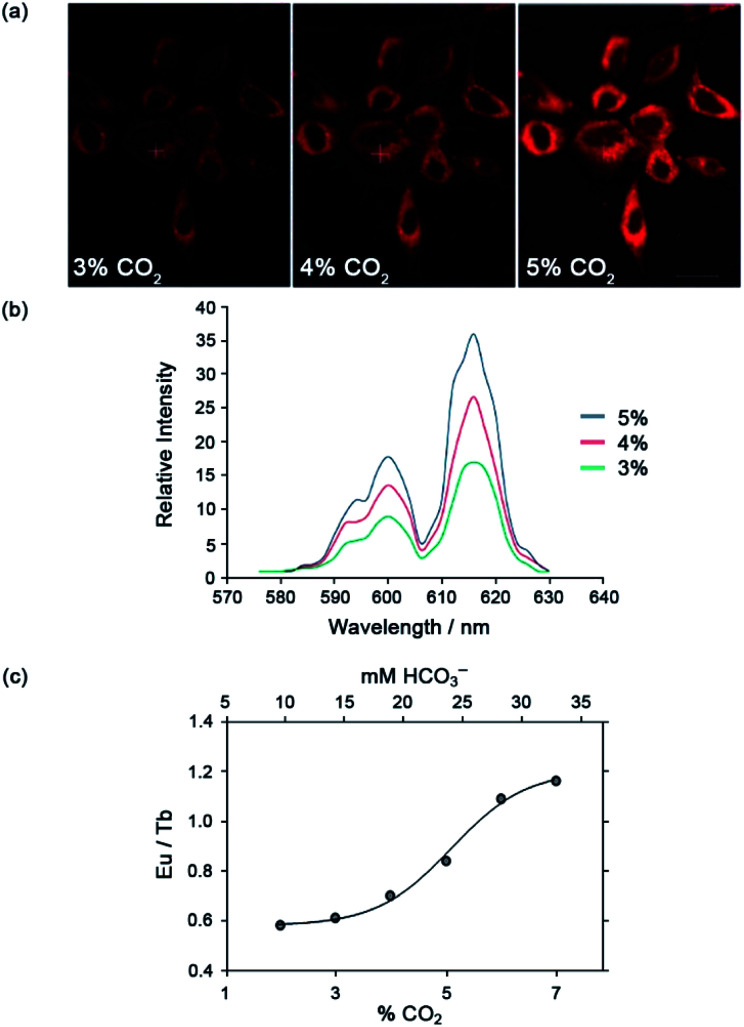
(a) Confocal microscopy images of HeLa cells, showing the mitochondrial region stained by [Eu-**33**]^3+^ under 3, 4 and 5% CO_2_ (1 h incubation, 20 μM complex, 30 min equilibration period between images); (b) variation of Ln(iii) emission intensity from hyper-spectral analysis of microscopy images for HeLa cells stained with [Eu-**33**]^3+^; (c) plot of Eu(iii)/Tb(iii) emission intensity ratio for [Ln-**34**]^3+^ (600–720 nm *vs.* 450–570 nm) as a function of pCO_2_, showing fit of experimental data for an effective *K*_d_ = 23.3 (±0.8) mM [HCO_3_^−^], observed in the mitochondria of NIH-3T3 cells. Reproduced (adapted) from ref. ^4^ with permission from Wiley-VCH, copyright 2012.

The Eu(iii) and Tb(iii) complexes were shown to co-localise in the mitochondria of several different cell types, enabling the steady-state concentration of HCO_3_^−^ to be monitored in living cells. The percentage of external CO_2_ was reduced incrementally from 7% to 2% and then increased back to 7%. The subsequent change in mitochondrial HCO_3_^−^ concentration was monitored by fluorescence microscopy, revealing a corresponding decrease in Eu(iii) emission intensity as pCO_2_ was reduced, and subsequent increase upon return to 7% external CO_2_ (Fig. 17).·The analogous Tb(iii) complex [Tb-**33**]^3+^ showed negligible changes in emission, thereby allowing ratiometric analysis of intracellular HCO_3_^−^ levels by monitoring the red/green (600–720/450–570 nm) emission ratio (Fig. 17c).

Subsequently, the Parker group examined the binding of HCO_3_^−^ to a series of highly emissive Eu(iii) complexes [Eu-**35**]^+^–[Eu-**39**]^+^ (Fig. 16), based on the smaller TACN macrocycle, bearing two strongly absorbing pyridylalkynylaryl chromophores.^75^ Each complex was found to have one coordinated water, with the exception of [Eu-**38**]^+^ (*q* = 0), which appears to self-associate in solution, *via* the remote carboxylate groups. Anion binding was examined in 50% aqueous methanol solution, revealing similar changes in emission spectra for HCO_3_^−^, citrate, lactate and benzoate, indicative of a common chelation binding mode. Monophosphate anions including HPO_4_^2−^, pTyr, pSer and pThr, produced a common change in emission spectra that differed to those observed for the chelating anions, characterised by appearance of a sharp, intense component within the ▵*J* = 2 band at 613 nm, attributed to a monodentate binding mode. Estimation of binding constants revealed similar affinities for HCO_3_^−^, citrate, lactate and monophosphate anions, in the range log *K*_a_ = 3–4. However, HCO_3_^−^ binding could be distinguished readily from the other anions by the large enhancement in the ▵*J* = 2 emission band, giving rise to a 30% increase in the ▵*J* = 2/▵*J* = 1 emission ratio.

Given that the concentration range of HCO_3_^−^ in human serum is 24–27 mM, around 10–20 times greater than other oxyanions (including phosphate, lactate and citrate),^2^ it was proposed that selective sensing of HCO_3_^−^ could be attained. Incremental addition of HCO_3_^−^ to a degassed solution of human serum containing complex [Eu-**37**]^+^, resulted in an approximately linear increase in the intensity ratio of the ▵*J* = 2/▵*J* = 1 bands, over the HCO_3_^−^ range 15–45 mM, with *K*_d_ = 37 mM. The ability of receptor [Eu-**37**]^+^ to signal HCO_3_^−^ in the presence of other biological anions is promising for the rapid quantification of HCO_3_^−^ in blood serum samples, offering the advantage of its higher inherent brightness compared to complexes of type [Ln-**34**]^+^, enabling lower probe concentrations to be used in analysis.

**Fig. 18:**
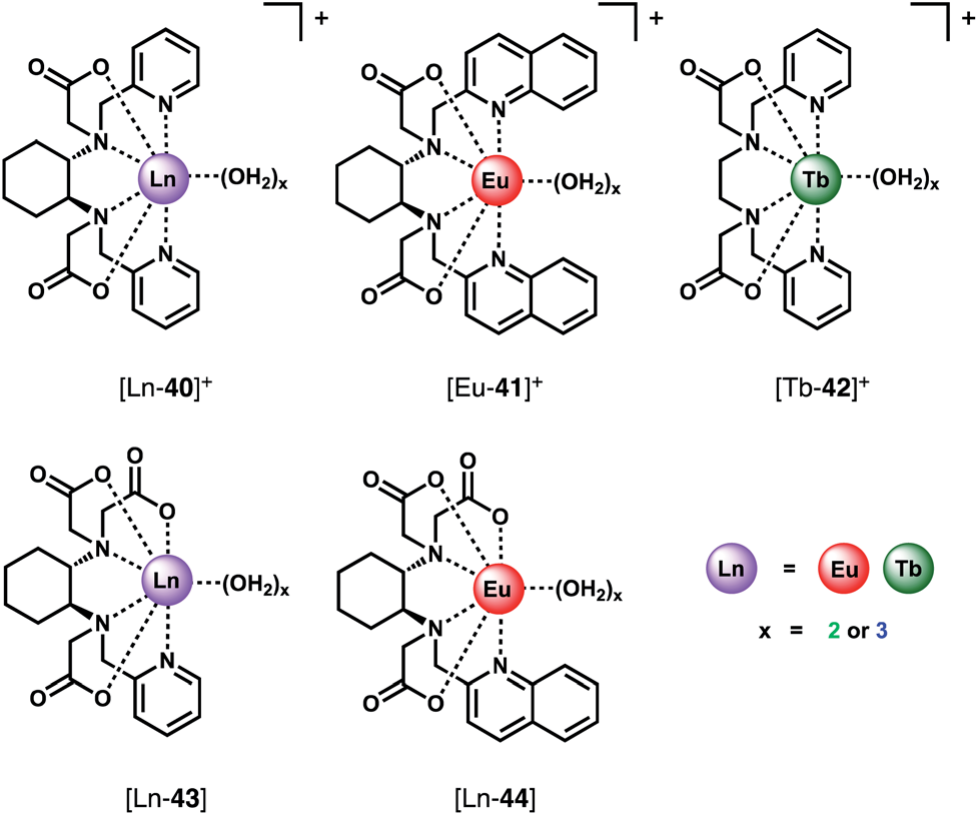
Series of Ln(iii) complexes of hexadentate ligands investigated as CPL probes for the detection of HCO_3_^−^.

The binding of a series of Eu(iii) and Tb(iii) complexes of chiral hexadentate ligands to HCO_3_^−^ was reported by Piccinelli and co-workers (Fig. 18).^76^ The complexes differ in their overall charge ([Ln-**40**]^+^, [Eu-**41**]^+^ and [Tb-**42**]^+^ are cationic, whereas [Ln-**43**]^+^and [Eu-**44**]^+^ are neutral), steric hindrance at the metal ion and lipophilicity of the heterocycles, which impact on the stability of the host–guest complexes. The hexadentate ligands form Eu(iii) and Tb(iii) complexes in water with varied stability (log *β* = 9.8–15.7), depending on the number of acetate donors. Each complex possesses either two or three inner sphere water molecules (*q* = 2.5).^77^ Addition of HCO_3_^−^ to the Eu(iii) complexes in water (pH 7.4) gave rise to an increase in intensity of the ▵*J* = 2 band and a small decrease in the ▵*J* = 0 band. Cationic complexes [Eu-**40**]^+^, [Tb-**40**]^+^and [Eu-**41**]^+^were shown to bind two HCO_3_^−^ ions with relatively high affinity, in the range log *K*_a_ = 4.62–5.94. In contrast, the neutral and less sterically hindered complexes, [Eu-**43**]^+^ and [Eu-**44**]^+^, bind one only equivalent of HCO_3_^−^ with lower affinity in the range log *K*_a_ = 2.06–3.11. In the latter cases, it is likely that the negative charge of the 1 : 1 adducts disfavours binding of a second HCO_3_^−^ ion. The high affinity of the cationic complexes for HCO_3_^−^ compared with those previously reported^4,75^ indicates their potential for sensing lower blood serum HCO_3_^−^ levels (*e.g.* below 23 mM),^78^ potentially aiding the diagnosis of metabolic acidolysis associated with kidney disease. However, while the authors note that [Tb-**43**]^+^ binds weakly to l-lactate (log *K*_a_ 1.3–1.45),^79^ selectivity over other anions (*e.g.* HPO_4_^2−^, citrate) or the potential interference from serum proteins was not investigated.

Recently, Sørensen and co-workers investigated the binding of HCO_3_^−^ to Eu(iii) complexes of classical DOTA (1,4,7,10-tetraazacyclododecane-1,4,7,10-tetraacetic acid) and DO3A ligands (Fig. 4a).^80^ The study highlighted the influence of buffer type, ionic strength and pH on anion affinity. As expected, [Eu–DO3A] bound HCO_3_^−^ in aqueous buffer, displacing the two coordinated waters, but was not selective for HCO_3_^−^ over other oxyanions including HPO_4_^2−^, lactate and citrate. The luminescence response of [Eu–DO3A] in the presence of 30 mM HCO_3_^−^ was also shown to be pH dependent, with the emission signal significantly increasing at higher pH (from 6.8 to 8.0). Bicarbonate titrations at different pHs confirmed that although the proportion of HCO_3_^−^ relative to other carbonate species does increase as the pH is raised from 6.8 to 8.0, the direct influence of pH on the binding event is much stronger, with binding constant increasing from *K*_a_ = 0.004 to 50 M^−1^. Importantly, such pH dependence suggests that HCO_3_^−^ sensors of this type can only operate in conjunction with a pH sensor (*i.e.* the pH of the solution must be known before the HCO_3_^−^ concentration can be accurately determined).

## Fluoride receptors

The binding of fluoride to Ln(iii) centres in water has attracted increasing interest in recent years. Receptors showing high selectivity for F^−^ could enable monitoring F^−^ levels in drinking water, which is important because excessive F^−^ levels can cause dental and skeletal fluorosis, acute gastric problems and kidney failure.^81,82^ The maximum concentration of F^−^ in drinking water is recommended to be 4 mg L^−1^ (210 mM) by the World Health Organisation, hence receptors with high affinity and sensitivity for fluoride are needed. Besides their potential environmental applications, Ln(iii) receptors that bind F^−^ have been very useful for studying the NMR and emission spectroscopic behaviour of paramagnetic Ln(iii) complexes and their supramolecular assemblies.

**Fig. 19:**
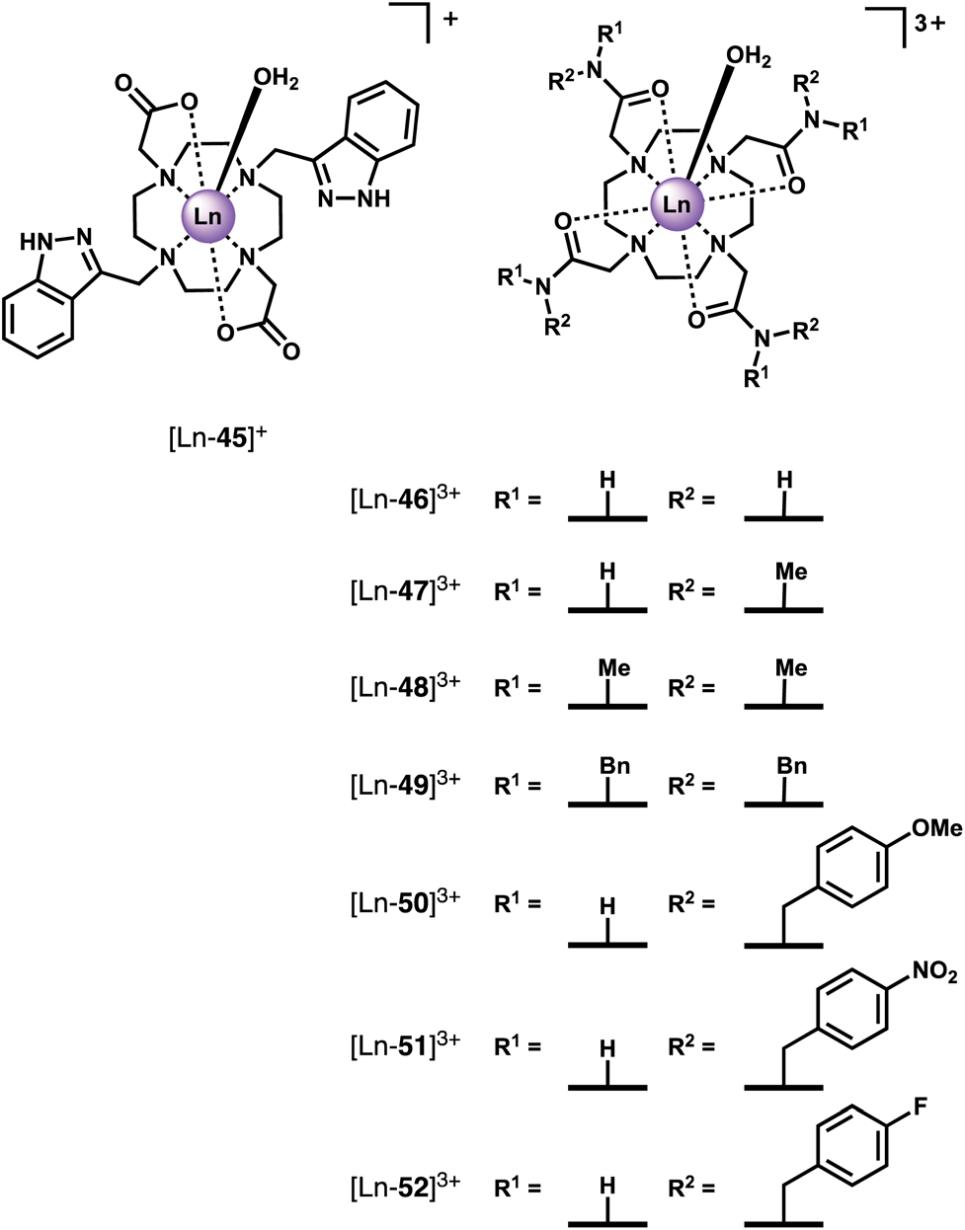
Structures of Ln(iii) complexes that bind and sense fluoride in aqueous solution.

One of the earlier examples of fluoride recognition involved a cationic Eu(iii) complex [Ln-**45**]^+^ (Fig. 19) reported by Charbonnière and co-workers, wherein F^−^ is sequestered in a supramolecular lanthanide dimer.^83^ [Ln-**45**]^+^ is based on cyclen with two pendant imidazole arms and two carboxylate arms. Upon the addition of F^−^, a sandwich-like structure forms involving a linear Eu–F–Eu bonding array, stabilised by π–π stacking of the imidazole heterocycles. Luminescence titrations revealed the strong cooperative binding in aqueous solution (log *β* = 13.0 ± 0.3). A 22-fold enhancement in emission intensity (*Φ* = 4.9%) was observed upon addition of F^−^, providing a highly sensitive response with a detection limit of 24 nM. The complex showed high selectivity for F^−^ over a range of anions including Cl^−^, Br^−^, HPO_4_^2−^, CH_3_CO_2_^−^ and HCO_3_^−^.

Using the erbium(iii) complex of the same ligand, the same authors reported the first evidence of molecular up-conversion in deuterated aqueous solution at room temperature.^84^ Low energy excitation of the Er(iii) absorption bands of [Er-**45**]^3+^ at 980 nm in D_2_O resulted in weak emission at 525, 550 and 650 nm, ascribed to Er(iii) centred transitions *via* a two-step excitation. The up-conversion signal was significantly increased by 7.7-fold upon addition of 0.5 equivalents of F^−^, owing to the formation of a supramolecular [(Er-**45**)_2_F]^+^ assembly. The large increase in emission for the dimer highlights the importance of the association of two Er(iii) metal centres in the up-conversion process and indicates an excited state energy transfer mechanism, in which a short internuclear distance is an important parameter.

In subsequent work, heterodimer formation was achieved using equimolar mixtures of Eu(iii) and Tb(iii) complexes of [Ln-**45**]^+^. In the presence of fluoride, selective excitation of the Tb(iii) metal (*λ*_ex_ = 488 nm) of the heterodimer resulted in Tb-to-Eu down-shifting energy transfer with 34% efficiency.^85^ Utilising this dual Eu/Tb emission (700/545 nm), ratiometric sensing of fluoride in water (pH 7.4) was achieved with a detection limit of 17.7 nM. Spectroscopic studies revealed the supramolecular dimers to be stable over a large pH range of 3–8.

Eu(iii) complex [Eu-**18**]^+^ (Fig. 8), developed by Butler, was found to bind F^−^ in water with high selectivity over other anions including Cl^−^, Br^−^, I^−^, HPO_4_^2−^, CH_3_CO_2_^−^, HSO_4_^−^, and NO_3_^−^.^86^ Addition of F^−^ to [Eu-**18**]^+^ resulted in a 1 : 1 host–guest complex (log *K*_a_ = 4.1 ± 0.1, 25 mM MES, pH 6), in which Eu–F coordination causes displacement of a single coordinated water molecule. In contrast to the work of Charbonnière, no evidence for a 2 : 1 supramolecular dimer was found by emission and NMR spectroscopy, or by mass spectral analysis. DFT studies suggested the 1 : 1 host–guest complex is further stabilised by two C–H⋯F hydrogen bonding interactions with the pendant quinoline arms. Fluoride binding induced a very large (9-fold) enhancement in Eu(iii) emission intensity, particularly within the Δ*J* = 1 band around 595 nm, which enabled quantification of fluoride in drinking water samples, within the environmentally relevant concentration range (20–210 μM).

Faulkner and co-workers synthesised a series of axially symmetric tricationic Ln(iii) complexes based on tetraamide DTMA ligands ([Ln-**46**]^3+^–[Ln-**52**]^3+^, Fig. 19) and investigated the displacement of the axially bound water molecule by F^−^ using a combination of NMR and emission spectroscopy.^87–89^ The study revealed dramatic changes in crystal field splitting upon F^−^ binding, which plays a significant role in determining the observed spectral properties of paramagnetic host–guest complexes.

In the presence of fluoride, the ^19^F NMR spectra of the diamagnetic complexes [Lu-**47**]^3+^ and [Y-**47**]^3+^ in D_2_O each showed the presence of two signals; one at −122 ppm corresponding to unbound F^−^, and a second signal at −74 and −58 ppm for the Lu and Y complexes respectively, corresponding to a F^−^ bound species. The ^19^H NMR spectrum of [Yb-**47**]^3+^ showed one (square antiprismatic) conformation in D_2_O (seven signals) and once F^−^ was added a new set of seven signals were observed.^87^ Exchange correlation spectrum (EXSY) experiments confirmed the original peaks and new set were in exchange. Interestingly, analysis of the EXSY cross peaks revealed that upon binding F^−^, the sign of the chemical shift of each proton changes, and the ordering of the peaks (from low frequency to high) is reversed. This change is due to a decrease in the second order crystal field coefficient, *B*_0_^2^ (to around 28% of its original value) and reversal of its sign upon binding fluoride. Further evidence for a decrease in magnitude of *B*_0_^2^ is given by changes in the fine structure of the emission spectrum of the [Eu-**47**]^3+^ complex, specifically the splitting of the Δ*J* = 1 band dramatically decreases, and two easily distinguishable peaks merge to form one apparent peak.^89^

The ^19^F NMR spectra of Ln(iii) complexes of **47** with added F^−^ revealed peaks for bound F^−^ with large shifts (*e.g.* −479 ppm for [Eu-**47**]^3+^), consistent with slow exchange of F^−^ on the NMR timescale. Titration experiments revealed relatively weak binding of F^−^ to the Eu(iii), Yb(iii) and Lu(iii) complexes, with 1 : 1 binding constants in the range log *K*_a_ = 1.0–1.9. These are smaller binding constants compared with those determined independently by Charbonnière and Butler, where F^−^ binding is stabilised through secondary hydrogen bonding and/or hydrophobic interactions with the heterocyclic arms.^89^

In subsequent work, the family of DTMA ligands was extended ([Ln-**48**]^3+^–[Ln-**52**]^3+^) to explore the effect of variations in amide structure and peripheral hydrophobicity on F^−^ binding.^88^ Binding constants, determined by ^1^H and ^19^F NMR and emission titration experiments, were in the range log *K*_a_ = 1.3–2.0. The incorporation of methyl groups into the ligand amides (from **46** to **48**) causes the fluoride affinity to decrease. Moreover, as the electron withdrawing nature of the benzyl substituent is increased (from OMe to F to NO_2_), the binding constant increases, indicating that the residual charge on the Ln(iii) centre strongly impacts on the binding constant. Interestingly, the introduction of hydrophobic benzyl substituents does not have a significant effect on the binding strength. However, the rate of F^−^ exchange (with the hydrated complex) was found to be slower for [Yb-**52**]^3+^, bearing hydrophobic benzylic groups, compared with [Yb-**47**]^3+^. Thermodynamic parameters extracted from linear Eyring plots showed Δ*H*^‡^ values for [Yb-**52**]^3+^ and [Yb-**47**]^3+^ are similar, implying electrostatic interaction between Yb^3+^ and F^−^ is independent of the ligand framework. However, Δ*S*^‡^ values were significantly different with [Yb-**52**]^3+^ having a more negative value, suggesting that the rearrangement of solvent in the vicinity of the metal centre with surrounding hydrophobic groups incurs a larger entropic cost during F^−^ binding, resulting in a slower rate of exchange.

These studies highlight how F^−^ binding to axially symmetric Ln(iii) complexes can change the sign and magnitude of the crystal field coefficient (which determines the magnetic susceptibility anisotropy), resulting in dramatic changes in NMR and luminescence spectra. The ability to predict, manipulate and control local crystal field will play an important role in the design of new responsive Ln(iii) receptors.

## Receptors for carboxylate anions

### Lactate receptors

Monitoring of lactate concentration is important for clinical diagnostics, since this anion is a specific biomarker for prostate and breast cancer, and elevated lactate levels are found in patients with Parkinson's disease.^90,91^ Seminal work by Parker demonstrated reversible binding of lactate to Ln(iii) centres using a combination of solution NMR studies and X-ray crystallography.^30,31^ The study revealed that lactate binding may occur *via* coordination of the CO_2_^−^ group (either in a monodentate manner or *via* a 4-membered chelate), or through a 5-membered chelate involving the OH and CO_2_^−^ oxygens (Fig. 4b). Subsequently, an anionic Eu(iii) complex with pronounced selectivity for lactate over citrate was devised, enabling lactate levels to be measured in the range 10 to 100 mM, in the presence of up to 100 mM citrate.^33,35^

**Fig. 20:**
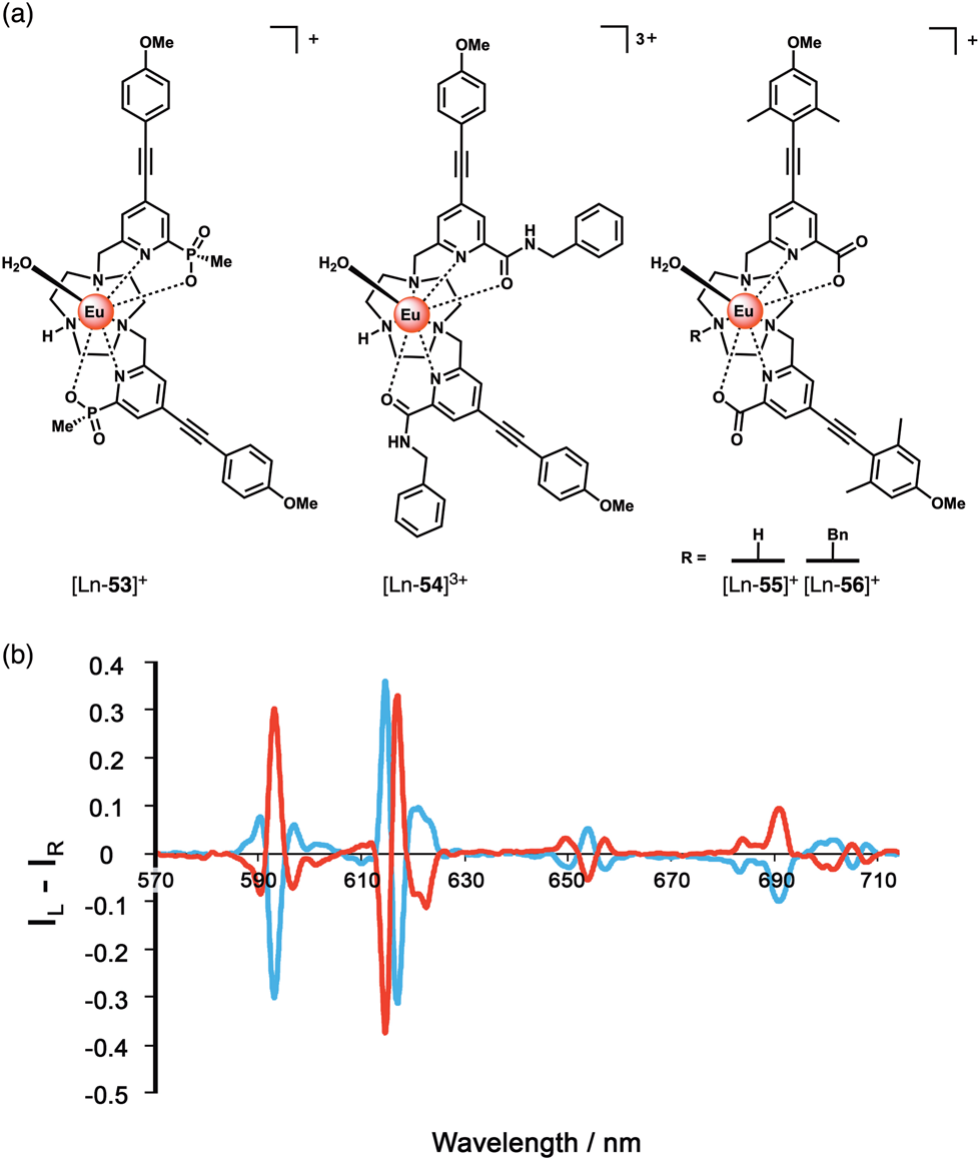
(a) Family of Eu(iii) complexes examined for their ability to signal α-hydroxy acids (*e.g.* lactate) by CPL spectroscopy; (b) mirror image CPL spectra of [Eu-**56**]^+^ following addition of *R*-(red) and *S*-(blue) lactate (*λ*_exc_ = 348 nm, 10 μM complex, 50 μM lactate, 295 K, MeOH). Reproduced from ref. ^92^ with permission from the Royal Society of Chemistry, copyright 2015.

More recent work has focussed on the development of Ln(iii) probes capable of sensing lactate by CPL spectroscopy (Fig. 20). The high sensitivity of CPL to subtle changes in Ln(iii) coordination environment is very useful for chiral sensing applications. A short series of Eu(iii) complexes (*q* = 1), prepared by Parker and co-workers from TACN ligands with two pyridylalkynylaryl antenna (bearing phosphinate, amide or carboxylate donors) were shown to bind chiral carboxylates including the α-hydroxy acids lactate and mandelate, forming 1 : 1 adducts.^92^ Anion binding was evaluated in a 1 : 1 water/methanol mixture due to limited water solubility of the Ln(iii) complexes. The strongest binding to lactate was observed for the tricationic bis-amide complex [Ln-**54**]^3+^, with log *K*_a_ = 4.57 (determined at pH 5.5 to eliminate interference from HCO_3_^−^), whereas the bis-phosphinate complex [Ln-**53**]^+^ showed the weakest affinity for lactate (log *K*_a_ = 2.76). However, the complexes showed stronger binding was observed for the bulkier and more hydrophobic anions, mandelate and cyclohexyl-hydroxyacetate, compared with lactate.

Analysis of luminescence lifetimes of the lactate adducts in water and D_2_O revealed *q* = 0 for [Ln-**53**]^+^ and *q* = 0.4 and 0.6 for [Ln-**55**]^+^ and [Ln-**54**]^3+^ respectively, indicating chelation of lactate to the latter two complexes *via* the OH and CO_2_^−^ oxygens, forming a 5-ring chelate, in which there is one O–H oscillator in the Eu(iii) coordination environment. For [Ln-**53**]^+^, lactate is proposed to bind *via* the CO_2_^−^ group only, due to the increased steric demand imposed by the bulkier phosphinate groups, supported by DFT calculations.^92^

The ability of the Eu(iii) complexes to signal lactate binding by the switching on of CPL was demonstrated. Addition of *R*- and *S*-lactate to each complex in methanol gave rise to mirror image induced CPL spectra (Fig. 20b). The *N*-benzyl complex [Ln-**56**]^+^ produced the strongest CPL signal, ascribed to helical alignment of the benzyl and two pyridyl groups, creating a more rigidified chiral structure. The *R* enantiomer gave rise to a common induced CPL signature across the series of Eu(iii) complexes, identified by the Δ*J* = 4 emission band around 700 nm. Notably, variations in the sign and magnitude of the CPL allowed the enantiomeric purity and absolute configuration of α-hydroxy acids to be estimated.

A chiral Tb(iii) complex [Tb-**40**]^+^ (Fig. 18) developed by Piccinelli and co-workers was found to bind lactate weakly in water.^79^ The ligand is based on a *trans*-1,2-diaminocyclohexane scaffold, which upon binding a Ln(iii) ion creates a dissymmetric environment, with *q* = 2. Lactate displaces the two inner sphere water molecules, signalled by an increase in Tb(iii) emission intensity, particularly the band at 546 nm, which plateaued after 100 equivalents of lactate (600 mM). DFT studies supported chelation of lactate to the Ln(iii) ion through the OH and CO_2_^−^ oxygens. Association constants revealed similar weak binding of l-lactate to the two enantiomers of [Tb-**40**]^+^ (log *K*_a_ = 1.3–1.45); however, the luminescence lifetimes for the two diastereomeric adducts were very different, pointing towards different geometries associated with the degree of distortion of their coordination spheres.

The binding of l-lactate induces a CPL signal for the racemic mixture of Tb(iii) complexes, due to the differing CPL activities of the diastereomeric adducts. In contrast, the achiral complex [Tb-**42**]^+^ did not show CPL activity, due to the flexible nature of the ethylenic group, allowing the interconversion between the two isomers compared with the cyclohexane derivative. The potential biological utility of [Tb-**40**]^+^ was demonstrated by detecting lactate levels in a commercial aqueous Ringer's solution, commonly used to treat metabolic acidosis. The authors note that for further biological application of the receptor, assessment of selectivity for lactate over other competitive anions (*e.g.* citrate, phosphate, HCO_3_^−^) is needed.

### Dicarboxylate anions

**Fig. 21:**
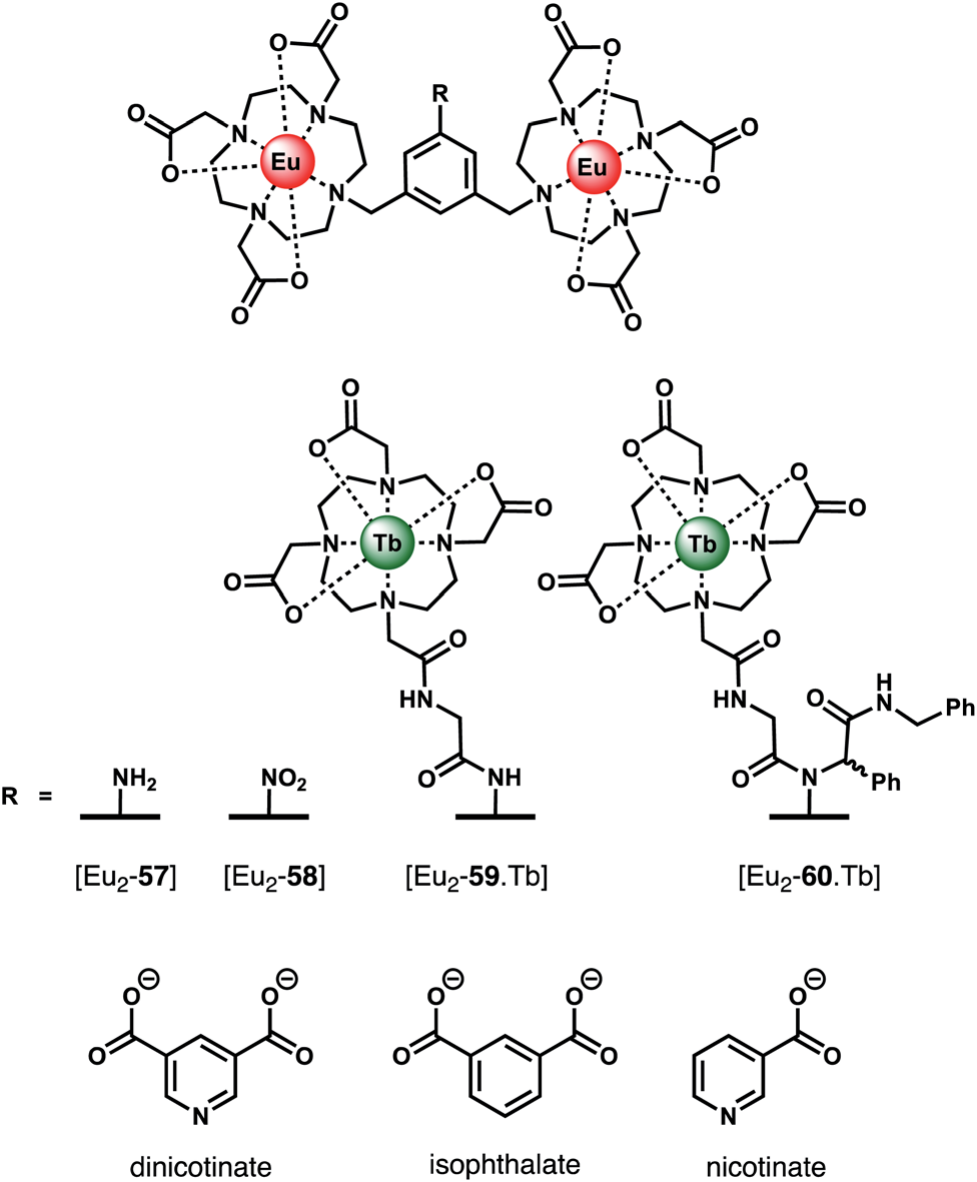
Di- and trinuclear Ln(iii) receptors capable of binding dicarboxylate anions (*e.g.* isophthalate) *via* a bridging mode.

Organic dicarboxylate anions (*e.g.* citrate, succinate, malonate and oxalate) represent an important class of biological substrates and attractive targets for anion sensing.^93,94^ Multiple carboxylate groups are found in close proximity in a variety of biomolecules, such as amino acids (Glu, Asp) and proteins. Early work by Gunnlaugsson and co-workers established the use of binuclear Ln(iii) complexes for the sensing of dicarboxylates, including terphalate, tartrate and malonate.^95,96^ More recently, Faulkner and Sørensen investigated the binding of aromatic dicarboxylate anions to a series of multinuclear Ln(iii) complexes (Fig. 21). Initially, a binuclear Eu(iii) complex [Eu_2_-**57**],^97^ comprising two DO3A units linked *via* a central *m*-xylyl scaffold, was shown to bind isophthalate in methanol forming a stable 1 : 1 adduct (log *K*_a_ = 5.1). Isophthalate bridges the two Eu(iii) centres, displacing solvent molecules. Next, the parent complex [Eu_2_-**57**] was derivatised to form two trinuclear complexes [Eu_2_-**59**·Tb] and [Eu_2_-**60**·Tb], each containing a bulky Tb(iii) reporter domain, attached *via* peptide and Ugi coupling methodologies, respectively.^98^ The impact of these remote bulky substituents on isophthalate binding was investigated in methanol. Complex [Eu_2_-**60**·Tb] binds most strongly to isophthalate (log *K*_a_ = 7.0), followed by [Eu_2_-**59**·Tb] (log *K*_a_ = 6.3), and the weakest binding to [Eu_2_-**58**·Tb] (log *K*_a_ = 5.1). In each case, isophthalate bridges both the Eu(iii) centres, and the increase in affinity is attributed to the increased steric bulk provided by the remote Tb(iii) domains in [Eu_2_-**59**·Tb] and [Eu_2_-**60**·Tb], which restricts conformational freedom around the Eu(iii) binding pocket, thus lowering the entropic cost for binding.

In subsequent work, the same authors quantified the entropic and enthalpic contributions to the binding of isophthalate and nicotinate to structurally similar binuclear complexes [Eu_2_-**57**] and [Eu_2_-**58**].^99^ Initially, anion titrations were conducted in methanol, in the presence of 1 mM LiOH, to ensure full deprotonation of the dicarboxylates and a constant ionic strength. The nitro derivative [Eu_2_-**58**] showed selectivity for isophthalate over dinicotinate, nicotinate and benzoate, with log *K*_a_ (298 K) = 4.3, 2.8, 3.4, and 2.7, respectively. Association constants for isophthalate and dinicotinate did not vary with temperature (293–313 K), indicating that in methanolic LiOH solution binding is disfavoured on enthalpic grounds but favoured entropically, consistent with the displacement of hydroxide increasing the overall disorder in the system.

The binding of nicotinate was examined further in different aqueous buffers (HEPES, BBS, PBS). Notably, the affinity of [Eu_2_-**57**] for dinicotinate in HEPES and BBS buffers was larger than that observed in methanolic LiOH solution, suggestive of competitive binding of hydroxide in the latter medium. Notably, no binding between [Eu_2_-**58**] and dinicotinate was observed in PBS buffer, due to the competitive binding of phosphate, whereas [Eu_2_-**57**] showed no effect of phosphate on the overall affinity.^100^ Other biological anions, including lactate (2 mM), pyruvate (0.3 mM) and citrate (0.01 mM), exhibited competitive binding to [Eu_2_-**58**] in BBS buffer (pH 8). In contrast to [Eu_2_-**57**], the solution structure of [Eu_2_-**58**] allows for competitive binding of anions that permit bidentate binding to a single Eu(iii) centre (*e.g.* phosphate, phthalate and pyruvate). This feature renders [Eu_2_-**58**] unsuitable for detecting dicarboxylates in biological systems. However, this study clearly illustrates how small variations in molecular structure of the host (or guest) can have dramatic effects on both affinity and selectivity between the Eu(iii) centres.

## Conclusions and outlook

This review highlights the powerful potential of luminescent lanthanide(iii) complexes for reversible anion binding and signalling in aqueous and biological media. Stable Ln(iii) complexes offer great scope for the design of selective anion receptors, in which the affinity and selectivity can be modulated by variations in the ligand structure and its conformational flexibility, steric hindrance at the metal centre, and the overall charge of the complex.

The emission spectra of Eu(iii) complexes are particularly sensitive to perturbations in the ligand field arising from anion binding to the metal centre. Modulation of the ligand field has been exploited to develop optical sensors for the selective detection of certain oxyanions (*e.g.* HPO_4_^2−^, HCO_3_^−^), the discrimination of nucleoside polyphosphate anions (*e.g.* ATP and ADP), and signalling of chiral anions (*e.g.*l-lactate) in competitive aqueous media. The interactions between host and anion are predominantly electrostatic in nature; however, the incorporation of secondary binding motifs within the ligand structure has proven successful for imparting additional levels of selectivity, through synergistic π–π stacking and/or hydrogen bonding interactions with the coordinated anion.

In certain cases, cell-penetrating Ln(iii) complexes have been created which localise to specific organelles of living cells and provide a fast and sensitive signal, which reports on fluctuations in anion concentrations (*e.g.*, HCO_3_^−^, ATP) in response to external perturbations of the environment. The most promising imaging probes provide a ratiometric read-out, either by comparing the intensity of two emission bands within a single Ln(iii) receptor or the Eu/Tb emission ratio of a dinuclear receptor. Such ratiometric probes provide an internal reference that facilitates calibration of the observed luminescence signal in living cells.

The development of anion-selective Ln(iii) receptors for monitoring enzyme activity is an emerging field, which exploits the ability of the receptor to signal the enzymatic formation or depletion of a target anion (*e.g.* ADP) accurately and rapidly, without perturbing enzyme reaction rates. The existence of commercial Ln(iii) bioassays in the form of labelled antibodies and proteins,^101^ means that appropriate instrumentation already exists for the creation of new Ln(iii)-based high-throughput screening assays, which can be adapted readily in drug-discovery research.

The key challenge remaining is to create Ln(iii) receptors which exhibit higher degrees of anion selectivity. In future receptor designs, it should be possible to integrate multiple recognition motifs within the ligand structure, which engage a target anion to generate a distinct host–guest complex structure. Stable Ln(iii) receptors with increased anion selectivity will open the door to new and improved assays and imaging probes required for biomedical and clinical research. The creation of near-infrared emitting complexes of Yb(iii) and Nd(iii) show promise for in-depth imaging of biological tissues in the NIR range (700–1200 nm),^102–104^ and their anion responsive properties should be explored further. Advances in receptor development should proceed with innovations in spectroscopy and microscopy instrumentation (*e.g.* fluorescence lifetime, CPL, and two-photon excitation microscopy)^105,106^ – such combined efforts will enable tracking of biological processes in living cells with higher spatial and temporal resolution. Innovations in these areas are eagerly anticipated in the near future.

## Conflicts of interest

There are no conflicts to declare.
